# Decoding topologically associating domains with ultra-low resolution Hi-C data by graph structural entropy

**DOI:** 10.1038/s41467-018-05691-7

**Published:** 2018-08-15

**Authors:** Angsheng Li, Xianchen Yin, Bingxiang Xu, Danyang Wang, Jimin Han, Yi Wei, Yun Deng, Ying Xiong, Zhihua Zhang

**Affiliations:** 1 State Key Laboratory of Software Development Environment, School of Computer Science, Beihang University, 100083 Beijing, P.R. China; 2 State Key Laboratory of Computer Science, Institute of Software, Chinese Academy of Sciences, Beijing, 100190 P.R. China; 3 School of Computer Science, University of Chinese Academy of Sciences, Beijing, 100049 P.R. China; 4 CAS Key Laboratory of Genome Sciences and Information, Beijing Institute of Genomics, Chinese Academy of Sciences, 100101 Beijing, P.R. China; 5 School of Life Science, University of Chinese Academy of Sciences, Beijing, 100049 P.R. China; 6 School of Mathematics, University of Chinese Academy of Sciences, Beijing, 100049 P.R. China; 7 School of Physics, University of Chinese Academy of Sciences, Beijing, 100049 P.R. China

## Abstract

Submegabase-size topologically associating domains (TAD) have been observed in high-throughput chromatin interaction data (Hi-C). However, accurate detection of TADs depends on ultra-deep sequencing and sophisticated normalization procedures. Here we propose a fast and normalization-free method to decode the domains of chromosomes (deDoc) that utilizes structural information theory. By treating Hi-C contact matrix as a representation of a graph, deDoc partitions the graph into segments with minimal structural entropy. We show that structural entropy can also be used to determine the proper bin size of the Hi-C data. By applying deDoc to pooled Hi-C data from 10 single cells, we detect megabase-size TAD-like domains. This result implies that the modular structure of the genome spatial organization may be fundamental to even a small cohort of single cells. Our algorithms may facilitate systematic investigations of chromosomal domains on a larger scale than hitherto have been possible.

## Abstract (web summary)

Accurate detection of TADs requires ultra-deep sequencing and sophisticated normalisation procedures, which limits the analysis of Hi-C data. Here the authors develop a normalisation-free method to decode the domains of chromosomes (deDoc) that utilizes structural entropy to predict TADs with ultra-low sequencing data.

## Introduction

In mammalian cells, the meter-long genome is folded into a complex three-dimensional (3D) configuration in order to fit inside the μm-size nucleus. The 3D architecture of the genome is essential to many processes in the nuclei^1–3^. The success of chromosome conformation capture and its variations in unraveling this architecture have stimulated the exploration of the 3D genome over the past decade^4^. With the accumulating data, the hierarchical configuration of the genome has started to emerge. Each chromosome can be largely partitioned into active and inactive compartments^5^, and these compartments may be further composed of domain structures, commonly named topologically associating domains (TADs) in mammals^6,7^. TADs have attracted much attention in the literature^8^, as they are found to constrain enhancer–promoter targeting in gene regulation^9–11^, correlated with the replication timing domain^12,13^, and conserved between cell types and species^6,8^. Moreover, the disruption of TAD boundaries may lead to the development of disease^14^ such as cancer^15^.

Several TAD detection algorithms have been developed^6–8,12,16–18^. TADs were first identified by a Hidden Markov Model, which detects regions with biased upstream and downstream chromatin interactions^6^. Filippova et al. introduced the notion of resolution-specific domains to identify TADs with a dynamic programming algorithm^19^. TADtree developed by Weinreb and Raphael identifies TADs with an empirical distribution of contact frequencies^20^. With the “arrowhead transformation” normalization method, Rao et al. identified multiple-scale TADs by a dynamic programming algorithm^21^. HiTAD developed by Wang and colleagues refined the definition of TADs by optimal separation of global chromatin interactions^12^. The Matryoshka algorithm proposed by Malik and Patro identified a consensus TAD hierarchy through domain clustering^22^, while 3DNetMod-MM^23^ and MrTADFinder^24^ borrowed the concept of graph modularity to identify hierarchical chromatin domains.

Even with the success of the aforementioned methods, several fundamental questions about TAD and its identification remain challenging. First, the global constraints that determine the hierarchical architecture of genomes remains to be elucidated. This question was addressed by setting a global object function while identifying TADs^12,23^. However, the object functions that the algorithms aimed to optimize are topological measurements, such as genomic distance^12^ and modularization of the genome^23^. Therefore, we still do not know what global constraint defines hierarchical TAD structures. Second, a method for how to determine the proper resolution for a given Hi-C dataset has not been established. Because of the sparsity and noisy nature of chromatin contacts, Hi-C data need to be divided into bins with a proper length before further analysis. The length of the bins (termed binsize) is a key to Hi-C analysis, and improper binsize setting may cause improper results or waste of sequencing data. However, the binsize is largely arbitrarily defined in current practice. Third, the question as to how to identify TADs reliably and stably with low-resolution Hi-C data has not been addressed. Almost all current algorithms require ultra-high coverage for TAD identification^6,21^. However, with the expanding applications of Hi-C technology, the requirement for massive sequencing depth has become an increasing hindrance to further expansion, particularly in the single-cell context. Finally, TAD is a statistical property of Hi-C data that was originally observed in bulk samples, which may be composed of millions of cells^6,7^. The genome architectures were found to be highly dynamic, with variations in the genome spatial structures from cell to cell, as indicated by single-cell Hi-C data^25–28^. Although pooling thousands of single cells' Hi-C data do reconstruct ensemble TADs^26,27^, it remains an open question how fundamental the TAD structure, or using a more general term, the “modular structure,” is for a small cell population.

Based on the structural information theory^29^, here we address the above questions by developing a TAD identification algorithm named decode the domains of chromosomes (deDoc). The structural information (or entropy) measures the uncertainty embedded in the dynamics of a graph. To minimize the structural entropy (SE) is an intuitionistic way to decode the essential structure of a graph, in which perturbations caused by random variation and noise have been reduced to a minimum. deDoc treats the Hi-C contact matrix as the connection matrix of the graphs and applies the SE to determine the 3D genomic architecture that has maximal certainty. We show that deDoc is distinguished from other state-of-the-art methods based on five outstanding features. First, the method is based on structural information theory^29^. Unlike most of state-of-the-art methods, which are mainly based on local contacting structures, deDoc is a graphic method seeking to extract a structure that minimizes the global uncertainty of the Hi-C graph. Second, deDoc works well with the raw Hi-C data, neither any normalization nor hand-choice parameters are needed. This excludes the effects of noise generated in the normalization or in the manual selection of parameters. Third, deDoc works well for highly sparse Hi-C graphs, which means deDoc is highly robust to the input data quantity. Fourth, we show that deDoc can be used for quantitatively determining the best binsize for a given Hi-C dataset. Last, we show that the megabase-size TAD-like domains can be unambiguously detected with pooled Hi-C data from 10 single cells using deDoc. This result implies that the modular structure of genome spatial organization may be fundamental and that data from a small cohort of single cells are sufficient for it to emerge.

## Results

### deDoc is an accurate tool for TAD detection

**Fig. 1:**
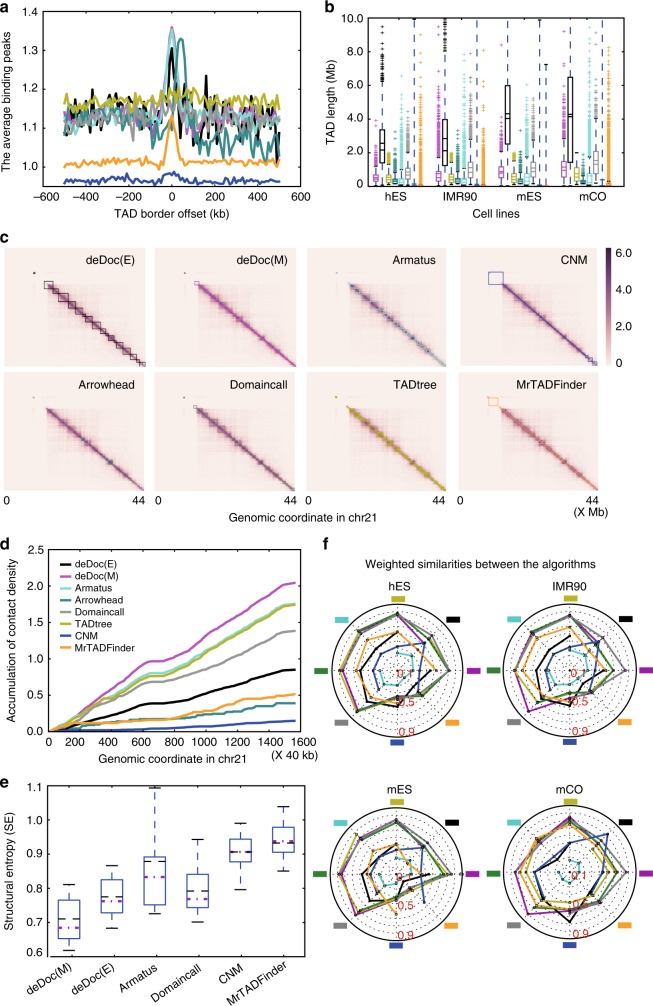
deDoc is an accurate TAD detection tool. The data analyzed in this figure was from Dixon et al.^6^. **a** deDoc identified border regions are enriched for CTCF bindings The plot represents ChIP-seq peaks with CTCF enrichment in human ES cells (hES). Each curve represents the result from an algorithm (color code for the algorithms in **d**). deDoc(M) and Armatus (magenta and turquoise, respectively) had the highest enrichment of CTCF-binding sites at their predicted TAD boundaries. **b** Box plots representing distributions of TAD lengths as predicted by each algorithm in four cell types. The mean and median of each prediction are indicated as dashed and solid lines, respectively. **c** Side-by-side comparison of the TADs predicted by each algorithm on chromosome 21. **d** deDoc identified more locally condensed TADs. Contact density is defined as the number of intra-TAD contacts divided by TAD length. The plot shows the cumulative contact density along chromosome 21 in hES cells. **e** TADs identified by deDoc have a lower structure information content. The boxplots summarized the structural entropies of TADs identified by the algorithms over all chromosomes in the hES cells. The means and medians are indicated by black and magenta lines, respectively. **f** Spider chart showing the similarities between the TADs predicted by the different algorithms. Each spoke represents a comparison of the weighted similarity (WS) between a reference algorithm (indicated as a colored square) and each of the other algorithms. Box plot quartiles, and the means and medians of each dataset are indicated by black and magenta lines, respectively. The ends of the up and down whiskers represent the highest and lowest datum or 1.5 interquartile range of the upper and lower quartiles, respectively

**Fig. 2:**
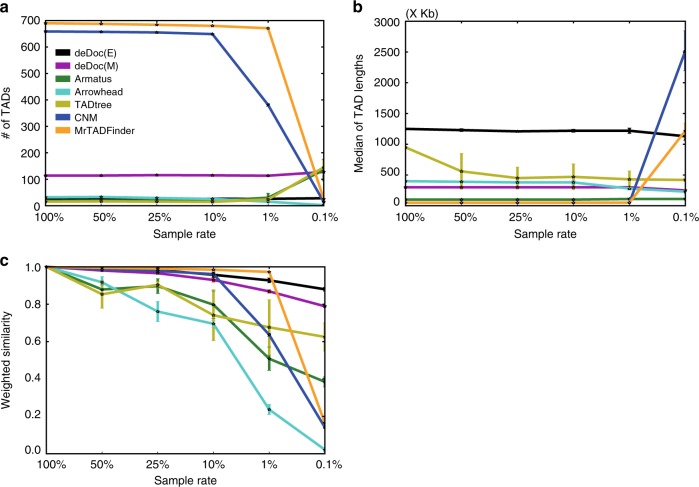
deDoc is a robust TAD identification algorithm. 50, 25, 10, 1 and 0.1% of the data from chromosome 22 in GM12878 cells (Rao et al.^21^) were sampled. **a** We compared the **a** the number of domains, **b** the lengths of the domains, and **c** weighted similarities of the TADs as identified using whole and down-sampled data. The error bars indicate s.d. from 50 replications of each experiment

**Fig. 3:**
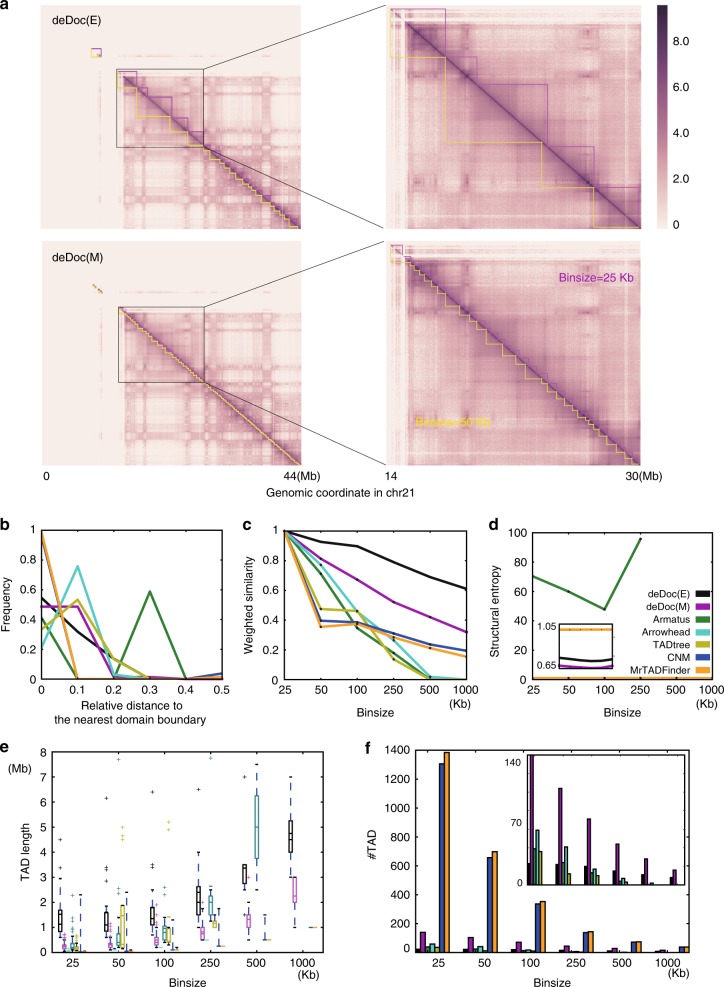
Hierarchical structure of genomes as detected by deDoc using different binsizes. **a** Heatmap of nested domains as predicted by deDoc(E) and deDoc(M) in human chromosome 21 are shown at the top and bottom panels, respectively. The predicted domains were highlighted in magenta and yellow sawteeth for binsizes of 25 and 50 kb, respectively. The distribution of **b** the relative distance to the nearest TAD borders, **c** the weighted similarities, **d** the structure entropies, **e** TAD lengths, and **f** the number of domains as predicted by the different algorithms using different binsizes. Each curve represents the result from one algorithm, and the color code for the algorithms are found in **d**. Zoom-in plots are embedded. Data from human GM12878 cells (Rao et al.^21^) were used to produce all plots in this figure. Box plot quartiles, and the means and medians of each dataset are indicated by black and magenta lines. The ends of the up and down whiskers represent the highest and lowest datum or 1.5 interquartile range of the upper and lower quartiles, respectively

To identify TADs from Hi-C data, we implemented the structural information^29^ based algorithms deDoc(2) and its variants, denoted as deDoc(E) and deDoc(M), respectively (Supplementary Figure 1a and Supplementary Table 1). The detailed algorithms can be found in the Methods section. Briefly, deDoc takes a Hi-C contact matrix as a weighted undirected graph and partitions it into subgraphs. The basic idea behind deDoc is to find an essential structure of a graph whose perturbations by random variation and noise has been reduced to a minimum. The structural information (or entropy) measures the uncertainty embedded in the dynamics of the graph^29^. Thus it is an intuitionistic way to decode the essential structure of a graph by finding a structure that minimize the SE. We show below that deDoc partitions the chromosomes into hierarchical domains that faithfully reveal the TAD structure. To assess deDoc, we compared it with five widely used algorithms, including Armatus^19^, TADtree^20^, Arrowhead^21^, MrTADFinder^24^, and Domaincall^6^, as well as with a classical graph modularity detection algorithm, denoted as CNM^30^ (Fig. 1, Fig. 2, Fig. 3,  Supplementary Figure 1, 2, 3, 4, 5, Supplementary Tables 1, 2 and 3, and Supplementary Data 1). The comparison was carried out with all algorithm operation with default parameters, except for MrTADFinder, which we set res = 2.875, as suggested by the original paper^24^.

deDoc can accurately detect TAD boundaries. Since there is no golden standard for TAD prediction available, we can only assess the accuracy of the algorithms indirectly. First, we examined the genomic features that was known to be associated with TAD boundaries. The CCCTC-binding factor (CTCF) is conservatively concentrated at TAD boundaries from fly to mammals^6,31–33^. Except for CNM, CTCF binding was, indeed, enriched in the predicted domain boundaries by almost all algorithms (Fig. 1a). We also checked the enrichment of all the histone modifications, chromatin-binding proteins, and transcription factors that have chromatin immunoprecipitation–sequencing data available in the ENCODE project in two cell types (hESC and mESC, in Supplementary Figure 3, 4, respectively). Similar to the previous report, housekeeping genes, H3K4me3, H4K20me1, and H3K36me3, are enriched in the predicted TAD boundaries^6^. Moreover, in all the cell types we examined (hESC, IMR90, mESC, and CO), the distribution of predicted TAD lengths by all the algorithms fall reasonably well within the normal length range (Fig. 1b).

Next, we compared the structural features of the predicted TADs. Visually, the TADs are dense squares in the Hi-C heatmaps (Fig. 1c). Structurally, the TADs are the genome regions within which intra-domain interactions are significantly enriched. We found that deDoc identified the TADs having the highest enrichment of intra-domain Hi-C contacts (Fig. 1d). Moreover, comparing to the other algorithms, we found that the TADs predicted by deDocs had the lowest SE (Fig. 1e). The SE measures the global uncertainty embedded in the dynamics of a graph^29^. The lower the SE of a domain structure, the more essential the structure is (see the section “Remarks on structural information (entropy)” in the Methods section).

Last, we compared the similarities between TADs predicted by different algorithms using a metric called weighted similarity (WS; see Eq. 9 in the Methods section). The pairwise comparison shows that, except for CNM, the detected domains were rather similar between deDoc and five other algorithms (Fig. 1f, Supplementary Figure 2). This indicates that the overall consistency of the domain boundaries between the predictions are generally very good and that the main difference between the algorithms might be the total number and lengths of the domains they return. Together, we have shown that deDoc can accurately partition genomes into the domains with the most significantly enriched characters for the currently understood TADs.

### deDoc performs well with ultra-sparse Hi-C data

To examine the performance of the algorithms with ultra-sparse input data, we composed a series of Hi-C datasets. Taking dataset of Rao et al.^21^ as the full dataset, we down-sampled to 50, 25, 10, 1, and 0.1% of the full data, respectively, to mimic different data volumes. First, we checked whether the numbers and lengths of the domains predicted with sparse input data is consistent to the original predictions with full data (Fig. 2a, b, respectively). deDoc algorithms were the single algorithms that did not substantially gain or lose predictions at 0.1% of full data volume. Although the lengths of the domains as predicted by TADtree, Arrowhead, and Armatus stayed almost unchanged at 0.1% of full data volume (Fig. 2a), the total number of domains was either substantially decreased (Arrowhead) or expanded (TADtree and Armatus, Fig. 2b). Domains predicted by both MrTADFinder and CNM contains only one or two bins when the input data are not too sparse (>1% of full data), but the domains became very large when the input data was only 0.1% of the full data.

Next, we asked how much of the domain structure could be faithfully predicted with lower input data volumes by the different algorithms (Fig. 2c). The structural faithfulness was measured as the WSs between the domains that were predicted with the full Rao et al.’s data compared to those predicted with down-sampled data. We found that deDoc algorithms were the only algorithms that faithfully predicted the domain structures with 0.1% of the full data (WS = 0.88 and 0.79 for deDoc(E) and deDoc(M), respectively, Fig. 2c). For CNM, Arrowhead, TADtree, and Armatus, the predictions remained only moderately faithful even when the input data volume were 10% of the full data (WS = 0.96, 0.70, 0.74, and 0.80, respectively). The four algorithms failed almost completely to make faithful predictions with 1% of full data. The WS of MrTADFinder’s prediction is 0.97 with 1% of full data; however, this is because the predicted domains are almost all single bins, and MrTADFinder failed to predict domains with high WS when the input data volume was 0.1% of full (WS = 0.16). Taken together, when the input Hi-C data is extremely sparse, e.g., 0.1% of Rao et al.’s data volume^21^, deDoc is the only algorithm able to consistently predict a reasonable number of domain structures with reasonable sizes.

### deDoc detects the hierarchical structures of the genome

We next asked whether deDoc could reveal the hierarchical structure of genomes. A natural way to reveal the hierarchical level of graph (chromosome) partitions is to trace back the hierarchical structure of its structure entropy minimalized coding tree. This relationship between the depth of the coding tree and the hierarchical level of TADs is determined by the definition of the coding tree (Fig. 1). By definition, the whole graph (chromosome) was set as the root of the coding tree, where the depth is 0; then the graph was roughly partitioned into a number of parts as the children of the root, where the depth is 1, and in each part, the partitioning proceeds iteratively with incremental depth till the parts are only single vertices or a given depth is reached. However, in deDoc, we chose depth 2 for deDoc(E), and deDoc(M) was then reapplied to the domains predicted by deDoc(E), which is equivalent to attain a depth of 3. We can therefore say that the domains predicted by deDoc(E) is at an upper layer of the hierarchical structure relative to those predicted by deDoc(M). This approach was chosen mainly based on the empirical observation that the partitioning at the depths of 2 or 3 is likely to be about the average size of canonical TADs. However, because there also exist smaller size domain types, e.g., sub-TADs^21^, and CTCF loop domains^34^, the domains at depth 3, may also be biologically relevant.

An alternative way to reveal the hierarchical structure of the genomes by deDoc would be to use different binsizes (Fig. 3). If domains, as identified by different binsizes, are in fact at different levels of the genome hierarchy, we should see most high-level domain borders overlapping with the borders at lower levels. By applying 25 and 50 kb binsizes to the data of Rao et al.^21^ as an example, we found that this was true for domains detected by both deDoc(E) and deDoc(M) (Fig. 3a). Further inspection showed that it was also true for most of the domain borders detected by deDoc(E) when compared to annotated compartment borders (Supplementary Figure 1c). For a genome-wide inspection of this assumption, we compared the distribution of relative genomic distances between domain borders using binsizes of 50 and 100 kb (Fig. 3b). For any given boundary as predicted using binsize of 100 kb, the relative distance was defined as “the distance to the boundary as predicted using binsize of 50 kb” over “the average length of the TADs predicted using binsize 100 kb, by the algorithm.” Comparing to the other algorithms, deDoc(M) has the highest frequency of predicted TADs that have shortest relative distance, i.e., the relative distance was <0.1, except for CNM and MrTADFinder. However, CNM and MrTADFinder are not comparable here, because most of the domains predicted by these two algorithms only contain a single bin at this resolution (Fig. 3e, f). This might be because CNM is not actually predicting TADs, and because the argument setting for MrTADFinder was not appropriate for this binsize (we used 2.875 as was recommended in the origin paper^24^). Next, it is obviously expected that higher WS values will be observed between domains sets that are hierarchically organized than between domain sets that are not. We consequently compared the WS values between the domains that were predicted using 25 kb bins and those predicted using larger bins (Fig. 3c). Comparing to other algorithms, deDocs had the highest WS in all binsizes tested. Last, we checked whether the domain structures as predicted using different binsizes kept their essentiality by the algorithms (Fig. 3d). The SEs of domains predicted by deDocs were consistently low and always the lowest in all the binsizes tested. In summary, deDoc can consistently partition genome into domains, and such partitioning under different resolutions reflects the hierarchical structure of the 3D genome.

### Using SE to determine binsize for a given Hi-C dataset

How to determine the proper binsize for a given Hi-C remains an open question. Intuitively, the best binsize should be the one that reliably captures the most essential structure of a given Hi-C dataset. Because SE characterizes the uncertainty of the high-dimensional hierarchical structure of a graph, and the minimization procedure of deDoc maximally reduces perturbations to a minimum, one would intuitively choose the binsize that minimizes the structural information to the fullest extent.

**Fig. 4:**
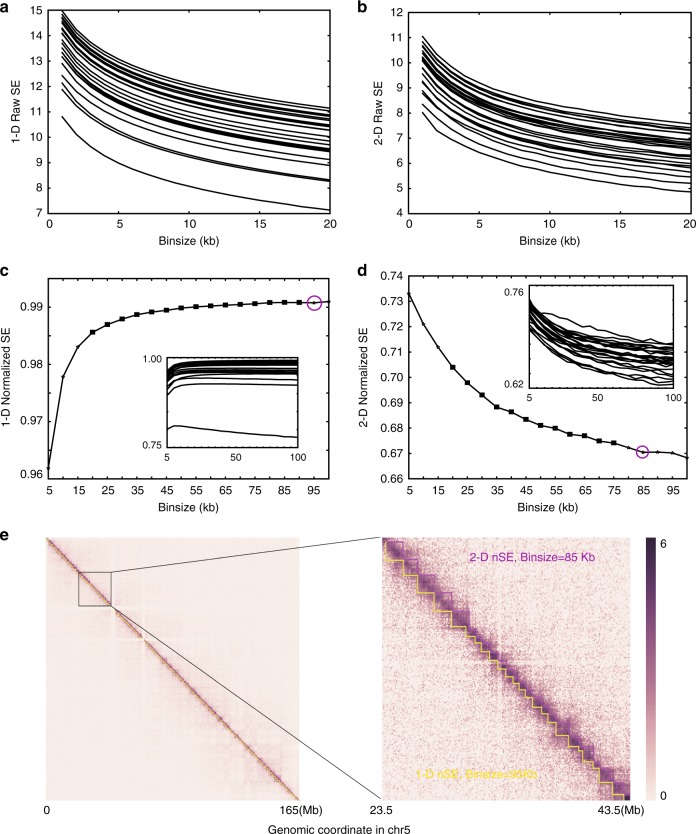
Using SE to determine the proper binsize for a given Hi-C dataset. Before normalization both **a** 1D-SE and **b** 2D-SE decrease in a near-monotonic manner with increasing binsizes in all 24 chromosomes. After normalization, both **c** 1D-SE and **d** 2D-SE differ far less with binsize for most of the binsize range. The main plots show data from chromosome 5, and the minimum stable binsizes are marked as magenta circles. The embedded plots show the curves for all 24 chromosomes. **e** Heatmap of the Hi-C data for chromosome 5 in hES cells taken from the data of Dixon et al.^6^ deDoc(M) predicted domains using binsizes of 85 and 95 kb are highlighted in magenta and yellow sawteeth, respectively. The two binsizes were determined by 1D-SE and 2D-SE, respectively, as the best resolution

However, the SE is not directly comparable between binsizes. We compared the 1D- and 2D-SE of the Hi-C graph from the dataset of Dixon et al.^6^, using binsizes from 10 to 100 kb. For all chromosomes, we found that both metrics decreased with increasing binsize in a near-monotonic manner (Fig. 4a, b), essentially because structural information is a metric that depends on the size of the graphs. In other words, SE is not directly comparable between binsizes before normalization. Because 1D-SE characterizes the uncertainty of the connectivity of the graph, we normalized the 1D-SE by dividing the SE by the total number of bins. The normalized 1D-SE (1D-nSE) is comparable between binsizes, except for very small binsizes (Fig. 3c). When the binsizes are very small, the graph becomes extremely sparse, and the corresponding 1D-nSE will be trivially low. In this case, we think that binsizes that correspond to trivially low 1D-nSE should not be chosen. The same reasoning demonstrates the validity of 2D-SE. To explain, we normalized 2D-SE (2D-nSE) by dividing 2D-SE by 1D-SE because 2D-SE characterizes the uncertainty of the high-dimensional hierarchical structure of the graph (Fig. 3d). The 2D-nSE is comparable between binsizes, again with the exception of very small binsizes (Fig. 3d) that should not be chosen as the corresponding 2D-SE values will be trivially high.

We chose the stable minimum binsize as the best resolution for TAD detection (see Methods). Both 1D-nSE and 2D-nSE could be used as the metric for binsize selection because the stable minimum binsizes, as determined by 1D-nSE and 2D-nSE, are close to each other. For example, we investigated a series of binsizes with 5 kb intervals from 10 to 100 kb, using the data of Dixon et al.^6^. For 19 out of the 24 chromosomes, both stable minimum1D-nSE and 2D-nSE exist (Supplementary Table 2). For 12 out of the 19 chromosomes, we found that the differences between the corresponding stable minimum binsizes identified by 1D-nSE and 2D-nSE, respectively, were within a size difference of 5 kb. Furthermore, even for chromosomes whose stable minimum binsizes identified by 1D-nSE and 2D-nSE by more than 5 kb, the TADs identified based on the binsizes by both methods were highly similar (Fig. 4e). We also found that the binsizes did not affect the TAD margins relative to the resolution (Supplementary Figure 5). It should be noted that a stable minimum binsize for chromosome chrY could not be identified by neither 1D-nSE nor 2D-nSE. This may be due to either the incompleteness of the dataset or the fact that current methods are approximation algorithms without the necessary precision required. Our results nonetheless demonstrate that both 1D-nSE and 2D-nSE are sound strategies for determining appropriate binsizes on autosomes. Intuitively, 2D-nSE might be regarded as better than 1D-nSE; however, 1D-nSE is significantly faster than 2D-nSE. In the present report, 1D-nSE was shown to be a sound strategy for binsize selection in most cases. Of course, for specific chromosomes, it is necessary to develop refined, or even higher-dimensional, strategies for detecting the most appropriate binsizes.

### TAD-like domains emerged from pooled single cells Hi-C data

Next, we asked whether deDoc can be used to address the question how fundamental the TAD structure, or, using the more general term “modular structure”, is for a small cell population. The hierarchical structure we explored above implies that a domain structure may exist at a higher level than canonical TADs. Given the capacity to faithfully identify chromosome domains with ultra-low input data volume (Fig. 2), we speculated whether deDoc might be able to reveal such higher-level domain structures with pooled Hi-C data from a small cohort of single cells. If this were possible, it would at least imply that the modular structure of genome spatial organization may be a fundamental characteristic and that data from a small cohort of single cells are sufficient for it to emerge.

**Fig. 5:**
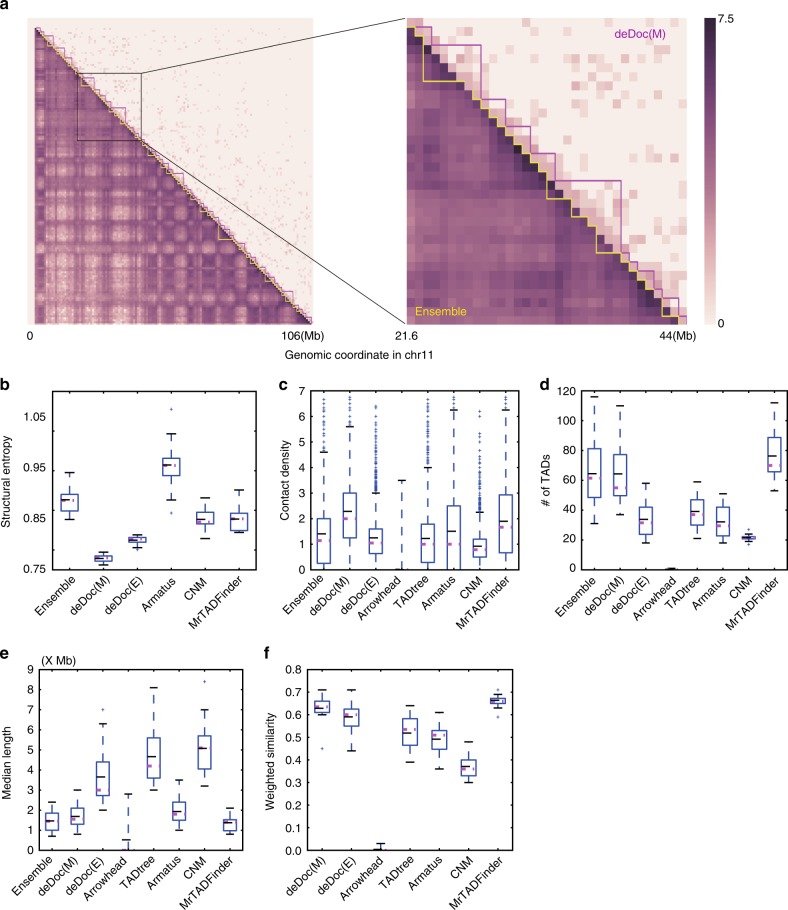
Megabase-size chromosomal domains can be detectable at the level of a few single cells. **a** Heatmap of ensemble TADs (magenta) and deDoc(M) domains predicted from data Hi-C pooled from 10 single cells (yellow). **b**–**e** Box plots of **b** the SEs, **c** intra-domain contact density, **d** the number, and **e** the length of the domains as predicted by the algorithms using the pooled Hi-C data. **f** WSs between the reference and the predicted domains. Box plot quartiles, and the means and medians of each dataset are indicated by black and magenta lines, respectively. The ends of the up and down whiskers represent the highest and lowest datum or 1.5 interquartile range of the upper and lower quartiles, respectively

To examine this possibility, we assessed the predictions of deDoc and six other algorithms on the pooled Hi-C data from 10 single cells^25^ (Fig. 5). The ensemble TAD structure, which was generated from the bulk sample and downloaded from the origin paper^25^, was used as the reference. First, the SEs of domains that were predicted by deDocs, CNM, and MrTADFinder was lower than the SE of reference (Fig. 5b), implying that the domains predicted should be at least as essential as those of the reference. Second, the intra-domain contact density of the domains that were predicted by deDocs, TADtree, Armatus, CNM, and MrTADFinder were comparable or higher than that of the reference (Fig. 5c). Third, the number of domains that were predicted by deDoc(M) and MrTADFinder are comparable to the reference (Fig. 5d). Fourth, the length of domains that were predicted by deDoc(M), Armatus, and MrTADFinder were comparable to the reference (Fig. 5e). Last, the WSs between the reference and the domains that were predicted by deDoc algorithms and MrTADFinder were much higher than the corresponding WSs predicted by the other algorithms (Fig. 5f). Collectively, the domain structures that were predicted by deDoc(M) and MrTADFinder with the pooled Hi-C data from 10 single cells were essential and sufficiently similar to the annotated ensemble TADs (Fig. 5a).

The domains that were predicted by deDocs and MrTADFinder with the pooled Hi-C data from 10 single cells may not necessarily be the canonical TADs, as the former were much larger. The canonical TADs were originally defined by the bulk Hi-C data^6,7^ and have average length about 500 k ~ 1 M. While the average lengths of the domains predicted by deDoc and MrTADFinder from the pooled Hi-C data from 10 single cells were 1.68 and 2.75 M, respectively, and thus much larger than canonical ones. We can presently think of explanations for this. One is that the TAD structure in the mouse CD41+ TH1 cells from which the single cell Hi-C data generated^25^ are indeed larger than canonical TADs, as their average length is 1.46 M (i.e., much larger than 500 k ~ 1 M of the canonical TADs). The other is that these domains might represent a higher hierarchical structure in the upper layer of the 3D genome organization. However, to determine which of the explanations is correct will need more sophistic assays.

## Discussion

In the present paper, we have reported a structural information theory^29^ based TAD detection algorithm, deDoc. The SE is defined over the coding tree of a graph by fixing and decoding the graph in a way that minimizes the uncertainty occurring in random walks in the graph. This means that the SE of the graph is the information embedded in the graph that determines and decodes the essential structure of the graph. The SE measures the information or uncertainty in situ wherever such uncertainty exists, without extracting any probabilistic distribution as in the Shannon entropy. Furthermore, the SE of a graph can be measured locally by fixing the vertices at which uncertainty is large. Thus the essence of deDoc algorithm is to fix the genomic loci at which the uncertainty of the structure is maximized. After fixing all the loci at which uncertainty is large, the algorithm deDoc identifies the essential structure that minimizes the uncertainty of the whole chromosomes.

deDoc is different from other state-of-the-art TAD prediction methods in the following four aspects. First, unlike most of other methods that are mainly based on local contacting structures of the chromosome, deDoc is a graphic method seeking the structure that corresponds to the minimal global uncertainty, i.e., the lowest structure entropy, in the Hi-C graph. This ensures that deDoc is an approach that detects the globally optimized structure of the genome. Recently, two modularity-based methods, named MrTADFinder^24^ and 3DNetMod-MM^23^, appeared in the literature. Although modularity is also a global property of the graphs, it is conceptually different from SE^29^. A detailed analysis of SE can be found in Li and Pan’s original paper^29^. Practically, the differences between deDoc and the two modularity-based methods are considerable. Both MrTADFinder and 3DNetMod-MM have arguments that need to be empirically determined, and correct settings of these may be critical for both algorithms. For example, the authors of 3DNetMod-MM recommend users to performing a full sweep of values for a range of possible settings. An additional problem for our analysis was that, because there are multiple arguments that need to be carefully set in 3DNetMod-MM, and not all of them have default settings, it was difficult to do fair comparison with other algorithms that had default settings^23^. Because of this, we did not include the 3DNetMod-MM in the comparison of the present work. We have included MrTADFinder in our comparison because it has only one argument that needs to be set, and this had a recommended value (2.875)^24^. We found, however, that with this recommended setting MrTADFinder performed dramatically different on different datasets, implying that the algorithm is heavily dependent on the setting of the arguments. In contrast, deDoc does not have any arguments and had a similar performance on all the datasets we tested.

Second, deDoc works well with raw Hi-C data without any normalization. This makes deDoc avoid the effects of noise generated in the normalization. Owing to the fact that the structure entropy allows deDoc to locally measure the uncertainty on site, data normalization is not unnecessary for finding the structure with minimum uncertainty. This feature of the structure entropy renders it markedly different from Shannon entropy, which measures only the global uncertainty based on a probabilistic distribution extracted from an observed structure. In summary, deDoc follows the principle of uncertainty minimization, which identifies the essential structure directly from the raw Hi-C data.

Third, deDoc works well for very sparse Hi-C graphs, which means that deDoc performs well even with small quantities of input data. For example, deDoc was able to predict TADs from only a small fraction, e.g., 0.1%, of uniformly and randomly sampled Hi-C data from Rao et al.’s data. In contrast, none of the other tested algorithms were able to make comprehensive predictions at this ultra-sparse Hi-C data level. Some algorithm may work with ultra-sparse data from certain Hi-C dataset, as did MrTADFinder on the pooled Hi-C data from 10 single cells; however, only deDoc consistently performed well on all datasets we tested. The ability of working in ultra-sparse Hi-C makes deDoc a potential powerful tool in a wide range of applications, e.g., the rare samples or samples for a large population.

Fourth, we showed that deDoc can be used for determining the best binsize for a given Hi-C dataset. There are three basic principles involved in the binsize determination. (1) The 1D- or 2D-SEs should be at a minimum, since these correspond to the best 3D structure that can be found in noisy data. (2) The 1D- and/or 2D-SEs should not be trivially low or exceedingly high, to avoid that the binsize will be unreasonably small or large. If the binsize is too small, the Hi-C contact matrix will be too sparse to identify any meaningful structures, while if the binsize is too large, the matrix will be too dense to distinguish meaningful domains from random variation. (3) The binsize should be at a stable minimum, which further ensured the non-triviality. However, the procedure we have proposed here for determining the best binsize may not work in all cases, and a more refined method thus needs to be developed. For example, the chromatin interaction peaks might be a potentially ideal metric for assessing the Hi-C data resolution. However, the problem with the interaction peaks is that, when the Hi-C resolution is not high enough, small binsizes will make the contact matrix too sparse to call statistically significant loops, while big binsizes will result in few loops that can be called from the matrix.

We applied deDoc to pooled Hi-C data from 10 single cells and revealed a megabase-size TAD-like domain structure^25^. However, this result does not indicate that there is a such TAD structure in each individual cell (Supplementary Figure 7). It has already been shown that the inter-cellular heterogeneity of chromosome 3D conformation is substantial^25^. It is even not known whether there exists TAD structures at the single-cell level, given the ultra-sparse Hi-C data that can be obtained from a single cell. This is also the reason why we do not claim deDoc works on Hi-C data from one single cell and instead asked whether domain-like structure would emerge if we pooled data from a small cohort of single cells? If this were the case, it would at least imply that the modular structure of genome spatial organization may be fundamental characteristic of the genome and might be detectable from a small cohort of single cells.

Lastly, there are two competing models as to what TADs actually represent. One model is concentrated on canonically defined TAD as actual intra-domain chromatin interactions in each individual cell. The alternative model is that TADs are the genome segments that may not have higher frequencies of intra-domain than inter-domain contacts in each individual cell; however, the intra-domain contacts are more consistent between cells, whereas inter-domain contacts are not. We want to emphasize that, although we observed domain-like structure by applying deDoc to pooled Hi-C data from 10 single cells, this result can neither prove nor disprove any one of the above models, and the understanding of the dynamics of the genome architecture at the single-cell level remains a major challenge to the field.

Taking together, the results of our experiments show that structural information is a powerful tool for revealing essential structures of the genome architecture. deDoc is a normalization-free method for TAD detection and its ability to reliably detect hierarchical TAD structures with ultra-low-resolution Hi-C data makes deDoc a promising tool to potentially expanding the field of 3D genome research by allowing the exploration of more cell types, tissues, and species.

## Methods

### Structural information theory

Given a Hi-C contact matrix $M = \{ m_{i,j}\}$ of a chromosome, we interpret the matrix *M* as a weighted graph (network) $G = \left( {V,E} \right)$, where the vertex set $V = \left\{ {1,2, \ldots ,n} \right\}$ contains all loci, i.e., bins, in the chromosome, the edge set *E* contains all interactions between the loci in *V*, and the Hi-C contact matrix *M* weighs the edge set *E*. We call the graph *G* the Hi-C graph of the chromosome. The problem of finding genome domains is now converted to a graph partition problem, i.e., finding a partition of the given graph that maximizes/minimizes an objective function. In deDoc, the objective function is the uncertainty of the positioning in random walks.

To measure the uncertainty, we employed the structural information theory recently developed by Li and Pan^29^. The basic idea behind this theory is to measure the information embedded in a graph *G*. Information content measurement was first introduced by Brooks^35^ and Shannon^36^ in communications. Brooks posed the question of how to define the information embedded in a graph so that it decodes the essential structure of the graph^35^, whereas Shannon asked whether or not there is a structural theory of information that supports communication graph analysis^36^. The question of quantification of structural information was recently addressed by Li and Pan^29^. We briefly review the structural information theory below. A verbal explanation about SE and a comparison to the Shannon entropy can be found in the Supplementary Note 1. The first question of the structural information theory is: how to encode a graph? Recall that the Huffman codes realize the optimum encoding of an unstructured alphabet Σ with a probability distribution *p*^37^. In the structural information theory, Li and Pan encode a graph using a partitioning tree^29^. In this manuscript, we call a partitioning tree a “coding tree” to facilitate a better understanding of the structural information in the context of genomics.

### The coding tree

A coding tree of a graph *G* is defined as a rooted tree *T* that has the following properties:

1. The root node *λ* is associated with the vertices set *V*. We termed *λ* the codeword of *V*, denoted as $c\left( V \right) = \lambda$, and termed *V* the marker of *λ*, denoted as $M\left( \lambda \right) = V$.
2. Every node $\alpha \in T$ is a codeword of a subset $X \subset V$, i.e., $c\left( X \right) = \alpha$ and $M\left( \alpha \right) = X$.
3. For every node $\alpha \in T$, suppose that $\beta _1,\beta _2, \ldots ,\beta _L$ are all the immediate successors of *α* in *T*; then all $M\left( {\beta _i} \right)$ are disjointed, and $M\left( \alpha \right) = \mathop {\bigcup}\nolimits_{i = 1}^L M \left( {\beta _i} \right)$.
4. For every leaf node $\gamma \in T$, $M\left( \gamma \right)$ is a singleton {*v*} for some vertex *v*, and for every vertex $x \in V$ there is a unique leaf node $\gamma \in T$ such that $M\left( \gamma \right) = \left\{ x \right\}$ and $c\left( x \right) = \gamma$.

### The structural information (entropy)

Let *T* be a coding tree of *G*, we then first define the structural information (or entropy) of a node in T. For every tree node $\alpha \in T$, if ${\mathrm{\alpha }} \,\ne\, {\mathrm{\lambda }}$, then the structural information of *α* is

1 $$H^T\left( {G;{\mathrm{\alpha }}} \right) = - \frac{{g_\alpha }}{{2m}}\mathrm{log}_2\frac{{V_\alpha }}{{V_{\alpha ^ - }}},$$

where *g*_*α*_ is the number of edges between the vertices in and not in $M\left( \alpha \right)$, *V*_*α*_ is the volume of vertices set $M\left( \alpha \right)$, i.e., the sum of the degrees of all the vertices in $M\left( \alpha \right)$, *α*^−^ is the immediate predecessor of *α*, and *m* is the sum of the edges, *sum*(*E*). The volume of *G* can be calculated as 2*m*. For an edge-weighted graph, the sum of edges *m* can be replaced with the sum of weights, $w = \mathrm{sum}\left( {w\left( E \right)} \right),$ in all the relevant formulas above.

The structural information (entropy) of a graph *G* given by the coding tree *T* is defined as

2 $$H^T\left( G \right) = \mathop {\sum }\limits_{\alpha \in T,\alpha \ne \lambda } H^T\left( {G;\alpha } \right).$$

Intuitively, $H^{\mathbf{T}}\left( G \right)$ is the number of bits required to determine the codeword in $c^{\mathbf{T}}({\mathrm{V}})$ of the vertex *v* that is accessible from random walk with stationary distribution in *G*.

In particular, for a natural number *k*, we define the *k*-dimensional structural information (entropy) of *G* as

3 $$H^K\left( G \right) = \mathrm{min}_T\left\{ {H^T\left( G \right)} \right\}$$

where *T* ranges over all the possible coding trees of *G* with height at most *k*.

When *k* = 1, the one-dimensional (1D) structural information is degraded to the following format:

$$H^1\left( G \right) = H\left( p \right) = H\left( {\frac{{d_1}}{{2w}}, \ldots ,\frac{{d_n}}{{2w}}} \right) = - \mathop {\sum }\limits_{i = 1}^n \frac{{d_i}}{{2w}}log_2\frac{{d_i}}{{2w}},$$

where *w* is the sum of weights, *d*_*i*_ is the weighted degree of vertex *i* in *G*, and $p_i = \frac{{d_i}}{{2w}}$.

By definition, $H^1\left( G \right)$ is the average number of bits required to determine the 1D codeword of the vertex that is accessible from the random walk with stationary distribution in *G*.

Finally, we define the structural information (entropy) of *G* as follows,

4 $$H\left( G \right) = \mathrm{min}_T\left\{ {H^T\left( G \right)} \right\}$$

where *T* ranges over all possible coding trees of *G*.

In Li and Pan’s original theory^29^, only the notion of *k*-dimensional structural information (entropy) was defined. We think the notion of structural information without specific dimensions, as defined herein, is also very interesting.

### Remarks for the structural information (entropy)

The structural information (entropy) *H*(*G*) in formula (4) has the following intuitions^29^:

1. *H*(*G*) is the minimum amount of information required to determine the codeword of a coding tree for the vertex that is accessible from a random walk with stationary distribution in *G*.
2. *H*(*G*) is the information embedded in *G* that determines and decodes a coding tree *T* of *G* such that the uncertainty in the codeword of *T* for the vertex accessible from random walk in *G* is at a minimum. An accompanying tree is defined as a coding tree *T* that has $H^T\left( G \right) = H\left( G \right)$. Consequently, an accompanying tree has minimum uncertainty in the partitioning of *G*. This means that an accompanying tree is the structure obtained from *G* by excluding the perturbation by noise and random variations to the largest extent. In another word, an accompanying tree represents the essential structure of *G*.
3. Given a graph *G*, the accompanying trees may not be unique. However, any such accompanying tree *T* must provide a reasonable interpretation of *G*. This is not surprising, because a syntax may have many different semantics.
4. How to compute the SE and an accompanying tree, or even me to determine the height of an accompanying tree *T* of *G*, is an important open question. In practice, we use only simple greedy algorithm to approximate the SE and the accompanying tree of the graph. In real world, 2D and 1D SE is good enough for us to decode the essential structure of a graph.

### The algorithm of deDoc

The algorithm deDoc(k) aims to find the coding tree *T* of height at most *k* with minimal $H^k\left( G \right)$. deDoc(k) is composed of two basic operators, merging and combining.

### The merging operator

Let $\alpha ,\beta \in T$ be two tree nodes, and $M\left( \alpha \right) = \left\{ {x_1,x_2, \ldots ,x_M} \right\}$ and $M\left( \beta \right) = \left\{ {y_1,y_2, \ldots ,y_N} \right\}$ the markers of *α* and *β*, respectively. We call *α* and *β* sister nodes, if both are immediate successors of the same node $\gamma \in T$, i.e., $\alpha ^ - = \beta ^ - = \gamma$. The merging operator, denoted as $Mg(T;\alpha ,\beta )$, is defined on the sister nodes *α* and *β* as follows:

1. Set $$M\left( \alpha \right) = \left\{ {x_1,x_2, \ldots ,x_M,y_1,y_2, \ldots ,y_N} \right\}$$.
2. Delete *β*.

We can easily derive the difference between the structural information of *G* given by the coding tree *T* and $T_{mg}(\alpha ,\beta )$ as

5 $$\Delta _G^M\left( {T;\alpha ,\beta } \right) = - \mathop {\sum }\limits_{\gamma {\it{\epsilon }}T:\alpha \subseteq \gamma \,or\,\beta \subseteq \gamma } \frac{{g_\gamma }}{{2m}}\mathrm{log}_2\frac{{V_\gamma }}{{V_{\gamma ^ - }}} + \mathop {\sum }\limits_{\delta {\it{\epsilon }}T:\alpha \subseteq \delta } \frac{{g_{\delta} }}{{2m}}\mathrm{log}_2\frac{{V_\delta }}{{V_{\delta ^ - }}}$$

where $T\prime = T_{mg}\left( {\alpha ,\beta } \right)$ is the coding tree obtained from *T* after the merging operation $Mg\left( {T;\alpha ,\beta } \right)$. If $\Delta _G^M\left( {T;\alpha ,\beta } \right) > 0$, we write $Mg(T;\alpha ,\beta ) \downarrow$.

### The combining operator

Let *α* and *β* be two sister nodes in a coding tree *T*, and ${\alpha}^{-} = {\beta}^{-} = {\delta} \; {\in} \; T$. We define the combining operator, denoted as $Cb\left( {T;\alpha ,\beta } \right)$, as follows:

1. Create a new tree node *ξ* with $M\left( \xi \right) = M\left( \alpha \right)\mathop{ \cup }\nolimits M\left( \beta \right)$ and $\xi ^ - = \delta$.
2. Let the branch $\left( {\delta - \alpha } \right)$ be $\left( {\xi - \alpha } \right)$, and let the branch $\left( {\delta - \beta } \right)$ be $\left( {\xi - \beta } \right)$, while maintaining the same order as that in *T*.

We can easily derive the difference between the structural information of *G* given by the coding tree *T* and $T_{cb}\left( {\alpha ,\beta } \right)$ as

6 $$\begin{array}{ccccc}\\ \Delta _G^C\left( {T;\alpha ,\beta } \right) = \hskip -45pt& H^T\left( {G;\alpha } \right) + H^T\left( {G;\beta } \right)\\ & - \left( {H^{T^\prime }\left( {G;\xi } \right) + H^{T^\prime }\left( {G;\alpha } \right) +H^{T^\prime }\left( {G;\beta } \right)} \right)\\ \end{array}$$

where ${\mathrm{T}}^\prime = {\mathrm{T}}_{cb}\left( {\alpha ,\beta } \right)$ is the coding tree obtained from *T* after the combining operation $Cb\left( {{\mathrm{T}};\alpha ,\beta } \right)$. If $\Delta _G^C\left( {T;\alpha ,\beta } \right) > 0$, we write $Cb\left( {{\mathrm{T}};\alpha ,\beta } \right) \downarrow$.

### The deDoc(k) algorithm

1. Initiation. Set $T_\lambda = V$ with $h\left( \lambda \right) = 0$, and for every $i \in \left\{ {1,2, \ldots ,n} \right\}$, define $T_{\lambda ^ \wedge \left\langle i \right\rangle } = \left\{ {v_i} \right\}$ with $h\left( {\lambda ^ \wedge \left\langle i \right\rangle } \right) = h\left( \lambda \right) + 1$.
2. Greedy merging. If there exist an $\alpha ,\beta \in T$ such that $(T;\alpha ,\beta ) \downarrow$, then

- choose *α* and *β* such that $\Delta _G^M\left( {T;\alpha ,\beta } \right)$ is maximized;
- set $$T = T_{mg}\left( {\alpha ,\beta } \right)$$;
- go back to step (2).

(3) Greedy combining. If there exist an $\alpha ,\beta \in T$ such that $Cb(T;\alpha ,\beta ) \downarrow$, then

- choose *α* and *β* such that $\Delta _G^C\left( {T;\alpha ,\beta } \right)$ is maximized;
- set $$T = T_{cb}\left( {\alpha ,\beta } \right)$$;
- go back to step (3).

(4) If there is some operation performed, go back to step (2), Otherwise, output the coding tree *T*, and terminate the program.

The algorithm deDoc(k) outputs a coding tree *T* of *G*. Clearly, the algorithm deDoc(k) works naturally on weighted graphs.

### Remarks on deDoc(k) algorithm

According to the description of algorithm deDoc(k), a domain of a chromosome is the marker of a node *α*, *M*(*α*) in the coding tree *T*. However, the *M*(*α*) is not necessarily a set of continuous loci in the chromosome. To guarantee that a domain is a set of continuous loci, we can modify the algorithm as follows:

For the merging operator $Mg\left( {T;\alpha ,\beta } \right)$, if $M\left( \alpha \right) = \left\{ {i,i + 1, \ldots ,j} \right\}$ and $M\left( \beta \right) = \left\{ {k,k + 1, \ldots ,l} \right\}$, where *j* < *k*, we may define $M\left( \alpha \right) = \left\{ {i,i + 1, \ldots ,l} \right\}$. For the combining operator with nodes $\alpha ,\beta \in T$, if $M\left( \alpha \right) = \left\{ {k\left| {i \le k \le j} \right.} \right\}$ and $M\left( \beta \right) = \left\{ {k\left| {s \le k \le t} \right.} \right\}$ for some *i*, *j*, *s*, *t* with *j* < *s*, then for the new node *ξ*, we define $M\left( \xi \right) = \left\{ {k\left| {i \le k \le t} \right.} \right\}$. We denote the algorithm with the modification above as deDoc(k)’. deDoc(k)’ is efficient, however, it is slower than the original deDoc(k).

Since every bin in the Hi-C matrix has many more interactions with its adjacent bins than with more distant bins, deDoc(k) cluster densely connected bins to form TADs that consists of contiguous bins. Our experiments showed that, with a few trivial exceptions, in all the dataset we tested TADs were composed of contiguous bins (Supplementary Table 1). The exceptions containing non-contiguous bins comprised about 1.7% and 2.7% of the domains predicted by deDoc(M) and deDoc(E), respectively. This fact verified the ability of deDoc to detect canonical TADs. Therefore, we simply use deDoc(k) in our research here and removed all the non-contiguous domains in the implements of deDoc(k).

### Time complexity of algorithm deDoc(k)

It can be easily derived that the time complexity of deDoc(k) is *O*(*n*^2^) when *k* = 2. However, the Hi-C contact matrix is nearly always sparse; therefore, we only need to consider time complexity under the condition of sparse graphs. When the graph is sparse, the time complexity becomes $O\left( {n\mathrm{log}^2{\it n}} \right)$ and $O\left( {n^2\mathrm{log}^2{\it n}} \right)$ for *k* = 2 and 3, respectively. Owing to the high complexity $O\left( {n^2\mathrm{log}^2{\it n}} \right)$ of our algorithm deDoc(3), we only considered algorithm deDoc(2), denoted as deDoc(*E*), in this study. To obtain smaller domains from deDoc(2) identified modules, we designed a modified version of deDoc(2), denoted as deDoc(M), which simply reapplies deDoc(2) to its identified modules.

### The similarity of two partitions

Let *X* and *Y* be two subsets of *V*, both of which consist of consecutive locations of a chromosome. We define the similarity of *Y* to *X* as follows:

7 $$S^G\left( {X,Y} \right) = \frac{{\left| {X\mathop { \cap }\nolimits Y} \right|}}{{\sqrt {\left| X \right| \cdot \left| Y \right|} }}$$

Suppose that $P = \left\{ {X_1,X_2, \ldots ,X_N} \right\}$ and $Q = \left\{ {Y_1,Y_2, \ldots ,Y_M} \right\}$ are two partitions of *G* such that each module *X*_*i*_ or *Y*_*j*_ is a consecutive set of vertices of *G*.

Then the similarity of each element *X*_*j*_, $j \in \left\{ {1,2, \ldots ,N} \right\}$ in *P* to *Q* is defined by the function $S_Q^P\left( j \right)$, as

8 $$S_Q^P\left( j \right) = \mathrm{max}_{i = 1}^M\left\{ {\frac{{\left| {X_j\mathop { \cap }\nolimits Y_i} \right|}}{{\sqrt {\left| {X_j} \right| \cdot \left| {Y_i} \right|} }}} \right\}.$$

Finally, the WS between *Q* and *P* is defined as the function $ws_Q^P$,

9 $$ws_Q^P = \frac{{\mathop {\sum }\nolimits_{j = 1}^N \left| {X_j} \right| \cdot S_Q^P\left( j \right)}}{{\mathop {\sum }\nolimits_{j = 1}^N \left| {X_j} \right|}}.$$

For any given algorithms *A* and *B*, if *P* and *Q* are the partitions of *G* found by algorithms *A* and *B*, respectively, then we define the WS between *A* and *B* as $ws_Q^P$, denoted as $ws_B^A$.

Remarks on the similarity of the two partitions can be found in Supplementary Note 2.

### Choice of binsizes

The intuition for binsize determination is to choose a binsize that minimizes the SE of the given Hi-C data. However, the number of vertices in *G* may have effects on the SEs. To make the SEs comparable between graphs with different numbers of vertices, we introduce the normalized structure entropy.

Let $G_k = \left( {V_k,E_k,W_k} \right)$, $k \in \left\{ {1,2, \ldots ,m} \right\}$ be the weighted graphs with different binsizes $b_1, \ldots ,b_m$, respectively. The 1D normalized SE (1D-nSE, for short) is defined as

10 $$H_{\mathrm{norm}}\left( {1,b_k} \right) = \frac{{H\left( {\frac{{d_1}}{{2w_k}}, \ldots ,\frac{{d_{n_k}}}{{2w_k}}} \right)}}{{\mathrm{log}_2 {\it n_k}}},$$

where *n*_*k*_ is the number of bins at binsize *b*_*k*_. The 2D normalized SE (2D-nSE, for short) is defined as

11 $$H_{\mathrm{norm}}\left( {2,b_k} \right) = \frac{{H\left( {2,b_k} \right)}}{{H\left( {1,b_k} \right)}}.$$

For a given Hi-C dataset, we choose the binsize that makes its associated Hi-C graph have the minimal normalized SE (nSE, for short) among all the stable binsizes in a series of incremental binsizes. A binsize *k* is considered stable if and only if both $H_{\mathrm{norm}}\left( {b_{k - 1}} \right) > H_{\mathrm{norm}}\left( {b_k} \right)$ and $H_{\mathrm{norm}}\left( {b_{k + 1}} \right) > H_{\mathrm{norm}}\left( {b_k} \right)$ hold.

### Sampling method

We randomly sampled raw reads from the tested Hi-C data.

### Code availability

The source code of the algorithm deDoc can be found at

https://github.com/yinxc/structural-information-minimisation.

### Data availability

The authors declare that all relevant data of this study are included within the article and its supplementary information. Public datasets used are summarized in Supplementary Table 1.

## Electronic supplementary material

Supplementary Information

Description of Additional Supplementary Files

Supplementary Data 1
