# Supplementary material for: Decoding topologically associating domains with ultra-low resolution Hi-C data by graph structural entropy
Source: Nat Commun. 2018 Aug 15;9:3265. doi: 10.1038/s41467-018-05691-7 (PMC6093941; doi:10.1038/s41467-018-05691-7)
Supplement: Supplementary file 1 — Supplementary Information [file 41467_2018_5691_MOESM1_ESM.pdf]

Supplementary Information

Decoding Topologically Associating Domains with Ultra-low resolution Hi-C Data by Graph Structural Entropy

Angsheng Li [1,3,4,*], Xianchen Yin[3,4], Bingxiang Xu[2,6,], Danyang Wang[2,6,],Jimin Han[4], Yi Wei[5], Yun Deng[6], Ying Xiong[7] and Zhihua Zhang[2,6,*]

[1] State Key Laboratory of Software Development Environment, School of Computer Science, Beihang University, Beijing, 100083, P. R. China

[2] CAS Key Laboratory of Genome Sciences and Information, Beijing Institute of Genomics, Chinese Academy of Sciences, Beijing 100101, P. R. China

[3] State Key Laboratory of Computer Science, Institute of Software, Chinese Academy of Sciences, Beijing, P. R. China

[4] School of Computer Science, University of Chinese Academy of Sciences, Beijing, P.R. China

[5]School of Mathematics, University of Chinese Academy of Sciences, Beijing, P.R. China

[6] School of Life Science, University of Chinese Academy of Sciences, Beijing, P.R. China

[7] School of Physics, University of Chinese Academy of Sciences, Beijing, P.R. China

* Correspondence should be addressed to Angsheng Li at: angsheng@ios.ac.cn or Zhihua Zhang at: zhangzhihua@big.ac.cn

Supplementary Note 1: The verbal explanation about structural entropy and a comparison to the Shannon entropy

The Structural entropy[1] is a metric to measure the information (uncertainty) embedded in a graph. The definition of Structural entropy was inspired by the Shannon Entropy[2], however, they differ in the following aspects.

First, the two Entropies were defined in different domains. The structural entropy was defined on graphs or structured data, while the Shannon entropy was defined on an unstructured probability distribution. Thus, the structural entropy measures the information (uncertainty) embedded in a graph, while the Shannon entropy measures the information in a probability distribution.

Second, the structural entropy is directly associated with the structure of a graph, while the Shannon entropy has no structure associated with. Given a graph *G*, and a coding tree *T* of *G*, which is intuitively a hierarchical partitioning of *G* , the structural entropy of *G* given by *T* is the uncertainty between the partitions (*T*) while random walking in *G*. In another word, we can calculate a structural entropy of *G* with any hierarchical partitions. On the other hand, for any given probability distribution, the Shannon entropy is purely a fixed number that measures the uncertainty of the distribution.

Thus, the goal of deDoc is to find a hierarchical partition (coding tree) of a given graph with minimal structural entropy. The algorithm starts from a trivial partition, which has each vertex as an individual domain. In each step, the algorithm greedily seeks an operation, i.e, to find two domains in the current partition to merge, such that the resulting partition has the maximally reduced the structure entropy over all possible combination of domain pairs.

More formally, the deDoc seeks to find the optimal partition as follows: Suppose that *P* is the current partition with *N* domains $X_1, X_2, \ldots, X_N$. We are going to merge two domains X_i and X_j in order to minimise the structural entropy. First, we notice that we only need to consider the two domains X_i and X_j with the edges between the two domains. For each such pair (i, j), we form

a new partition Q_{ij} consisting of $\mathrm{X} = X_i \cup X_j$ and all the other domains of *P*. Let $\Delta_{ij} = H^P(G) - H^{Q_{ij}}(G)$. By the definitions of $H^P(G)$ and $H^{Q_{ij}}(G)$, Δ_{ij} contains only the values associated with the two domains X_i and X_j, so that Δ_{ij} is locally computable. The algorithm deDoc then chooses (i_0, j_0) such that $\Delta_{i_0 j_0}$ is the maximum of Δ_{ij} for all possible *i* and *j*. Then we execute the operation of merging X_{i_0} and X_{j_0}.

Supplementary Note 2: Remarks on the similarity of the two partitions

First, the predictions made by different algorithms have huge differences in TAD sizes, something which has also been noted in the literature. Thus, a good metric should ideally also consider such domain size effect. However, as far as we know, most of the simple symmetry metrics do not or are unable to take into account the domain size effect. For example, the Jaccard Index, which was defined as the ratio between the size of the intersection and the size of the union of interactions and TAD boundaries called in different replicates. The size of the union or the size of the intersection in the definition refers to the number of elements in each set, not the actual size (length) of the domains. Thus, it is a good metric for loop set or single domain comparisons, but it may not be the best for comparisons between partitions. Another example that we can define a metric is the following: Given a partition *P*, for every pair (i, j) of vertices *i* and *j*, we define $a_{ij} = 1$ if *i* and *j* are in the same domain *X* in *P*, and 0 otherwise. This represents the partition *P* as an 0/1 vector $\mathbf{V_P}$. Suppose that *Q* is another partition, for which we define a similar vector $\mathbf{V_Q}$. We then use the distance between $\mathbf{V_P}$ and $\mathbf{V_Q}$ to define the similarity between *P* and *Q*. In this way, we may use different measures of distances between the two vectors, for instance, norm L_1, norm L_2, etc. A similarity defined in this way is symmetric. However, this similarity does not consider the balance of the size of domains and the size of intersections, and is therefore not suitable for comparisons between partitions.

Second, we do not think that a simple symmetry metric may bring us significant new knowledge. For example, we could define $ws(P,Q)$ to be minimum(s) of ws_Q^P and ws_P^Q. We did the same analysis as shown in Figure 1F using this symmetry metric $ws(P,Q)$ and found almost identical pattern as for ws_Q^P did (Supplementary Figure 8). With the ws_Q^P, we found that except for CNM and Arrowhead, the detected domains were rather similar between deDoc and the four other algorithms. The only difference is the comparison to Arrowhead. However, we argue that that is another reason why should we choose ws_Q^P, as detailed in the following paragraph.

Third, a simple symmetry metric may even introduce false assessment, such as in the case where a partition is not complete to a graph, i.e. a large portion of vertices are not included in the partition. In the above case, the reason why $ws(P,Q)$ between the predictions of Arrowhead and deDoc (and all the other algorithms) is so low is that the Arrowhead does not partition the whole genome completely (Figure 1C). Only about 17%, 18%, 15% and 7% of whole genome region were predicted as TADs by Arrowhead in hES, hIMR90, mES, and mCO cells, respectively. In other words, there are huge gaps in the genome which, however, were marked as unknown by Arrowhead. Therefore, when one partition is not complete to the genome, the $ws(P,Q)$, and any other metric that does not consider domain size and number effects, will report a rather low similarity. However, comparing the actual domains predicted by the algorithms, they share most of the boundaries, suggesting the predictions should be regarded as similar, and has thus been reported correctly using ws_Q^P (Figure 1F).

Last, when the two partitions are both complete**,** we argue that although ws_Q^P is not strictly symmetric, it is sufficiently similar between ws_P^Q and ws_Q^P.

Validation for similarity when the two partitions are complete.

1. The similarity $s^G(X,Y)$ in Equation 7 satisfies the following properties:

1) It is symmetric, that is, $s^G(X,Y) = s^G(Y,X)$;

2) If $X = Y$, then $s^G(X,Y) = 1$;

3) If the size of $X \cap Y$ is small, relative to the sizes of *X* and *Y*, then $s^G(X,Y)$ cannot be large;

4) If *X* is small and *Y* is large, then $s^G(X,Y)$ cannot be large;

5) If $s^G(X,Y)$ is large, then *X* and *Y* are similarly large, i.e., the sizes of the two sets are similar, and the intersection of *X* and *Y* is large relative to the sizes of *X* and *Y*.

Property 5) ensures that if $s^G(X,Y)$ is large, then *X* and *Y* are similarly large and the intersection of *X* and *Y* forms a major part of both *X* and *Y*, implying that if *X* is a meaningful domain, *Y* is a similarly meaningful domain.

2. The similarity function S_Q^P in Equation 8 is not symmetric between *P* and *Q*. It assumes that *P* is a “standard”, e.g., a ground-truth partition. The metric measures how similar *Q* is to *P*.

3. The weighted similarity ws_Q^P in Equation 9 is the average score of similarity by s_Q^P in Equation 8, so it is not symmetric.

4. The weighted similarity ws_Q^P satisfies the following properties:

1) If ws_Q^P=1, then ws_P^Q=1

2) If ws_Q^P is large, then there is a sufficiently large set *S* of domains in *P*, such that for any domain *X* in *S*, there is a domain *Y* in *Q* such that $s^G(X,Y)$ is high. This implies that the intersection of X and Y is large, and the *X* and *Y* are of similar sizes. Moreover, this similarity property holds for *Q* as well.

3) Because of 2), we have that if ws_Q^P is large, then so is ws_P^Q.

4) The arguments above demonstrate that although ws_Q^P is not strictly symmetric, ws_P^Q and ws_Q^P are sufficiently close to each other.

Supplementary Note 3: DeDoc is an easy-to-use and fast tool for TAD detection

The deDoc is an easy-to-use tool. First, it does not require normalisation for input data. It is well known that Hi-C experiments are always subject to systematic bias, which, in turn, can seriously influence subsequent data analysis [3]. Many normalisation methods have been proposed in the literature [3,4,5], however, it remains empirical in Hi-C data analysis to choose a proper way to perform normalisation. Although normalisation is useful in some cases, it unavoidably introduces new noise that must be taken into account, and it fills in gaps in the entries which hugely increases the time complexity of the detecting algorithms. Except for CNM, normalisation of the input data is required for almost all current algorithms, and there is a substantial risk the results are affected by improper normalisation methods [6]. It thus appears that the optimal strategy would be that the algorithm only uses the original data without normalisation (Supplementary Table 3). Because structural information theory does not require normalisation to find the minimum uncertainty[1], the deDoc algorithm takes the raw Hi-C matrix as input, and identifies the essential structure from the raw Hi-C data directly. Second, deDoc needs no setting of arguments. Thus, these two features make deDoc very easy to use.

The deDoc is also fast. The time complexity of deDoc and the modularity-based CNM method are nearly linear when input data are sparse, which is always the case for Hi-C data (Supplementary Table 3). We tested the speed of the algorithms. It took 72, 68, 50, 86, 3315, 4546 and 2220 minutes for deDoc(M), deDoc(E), CNM, Armatus, Arrowhead, MrTADFinder and TADtree, respectively, to finish TAD calling for the Hi-C data of chromosome 21 in GM12878 cells with 1kb binsize from Rao et al[4] (CPU: Intel Xeon 5160 at 2.30GHz, and memory: 1024GB). Thus, deDoc, CNM and Armatus are orders of magnitude faster than the others. Taken together, this shows that deDoc is an accurate, easy-to-use, and fast tool for TAD detection.

Dataset	Figure,Table	Reference	URL
hES, hIMR90, mES, mCO	Figure 1, Supplementary Data 1	Dixon, J.R. et al[7].	http://www.ncbi.nlm.nih.gov/geo/query/acc.cgi?acc=GSE35156
GM12878	Figure 2,3, Supplementary Figure 1(a), 2, 6	Rao, S.S. et al[4].	http://www.ncbi.nlm.nih.gov/geo/query/acc.cgi?acc=GSE63525
hESC_HindIII	Figure 4, Supplementary Figure 5, Supplementary Table 2	Dixon, J.R. et al[7].	http://www.ncbi.nlm.nih.gov/geo/query/acc.cgi?acc=GSE35156
mouse CD41+ TH1 cells	Figure 5, Supplementary Figure 7	Nagano, T. et al[8].	http://www.ncbi.nlm.nih.gov/geo/query/acc.cgi?acc=GSE48262
GM06690	Supplementary Figure 1(b), 1(c), 1(d)	Lieberman-Aiden, E. et al[9].	http://www.ncbi.nlm.nih.gov/geo/query/acc.cgi?acc=GSE18199
ChIP-seq	Figure 1a, Supplementary Figure 3,4	NA	https://www.encodeproject.org/

Supplementary Table 1. Public data used in this paper.

	1D-SI	M-SI
chr1	75	80
chr2	90	55
chr3	100	90
chr4	95	90
chr5	95	85
chr6	100	85
chr7	90	65
chr8	95	95
chr9	75	90
chr10	100	95
chr11	95	95
chr12	80	80
chr13	95	85
chr14	90	95
chr15	95	95
chr16	85	90
chr17	70	85
chr18	80	95
chr19	65	80
chr20	55	85
chr21	90	95
chr22	75	90
chrX	100	95
chrY	100	80

Supplementary Table 2. The best binsizes found for Dixon et al's Hi-C data.

Algorithms	Time complexity	Input data	Reference
deDoc(M)	$O(nlog^2n)$	Raw data	
deDoc(E)	$O(nlog^2n)$	Raw data	
CNM	$O(nlog^2n)$	Raw data	Clauset, et al[10]
Armatus	$O(n^2)$	Normalised	Filippova, et al[11]
TADtree	$O(nS^5)$	Normalised	Weinreb and Raphael[12]
Arrowhead	$O(n^2)$	Normalised	Rao, et al[4]
Domaincall	NA	Normalised	Dixon, et al[7]
MrTADFinder	NA	Normalised	Koon-Kiu Yan and Mark Gerstein[13]

Supplementary Table 3. Time complexity of the algorithms. In the Time complexity column, n and S denote the number of bins and the maximum domain size, respectively.

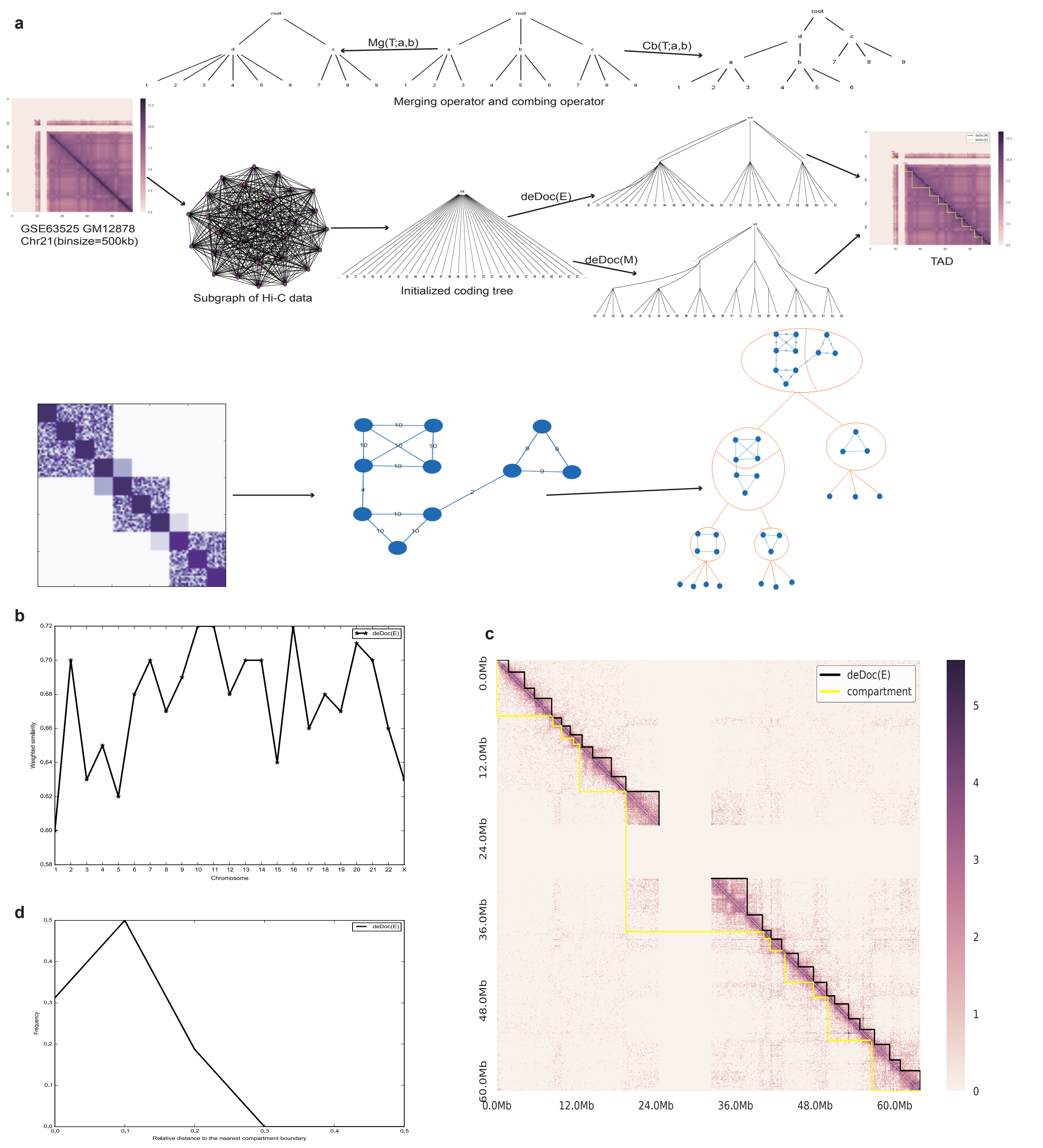

Supplementary Figure 1. The algorithm of deDoc. **a**, A cartoon demonstrating how the deDoc works. In the upper panel, it is a real data from GSE63525 in human GM12878 cells. Hi-C data from chr21 was first converted into a weighted fully connected graph. A trivial initialized coding tree was formed. By iteratively apply merging and combining operators, which showed on top, to seek minimal structure entropy, the final coding tree represents the detected domain structure. In the down panel, it is a cartoon showing a toy data, the basic idea of deDoc is to convert a domain prediction problem into a graph partition problem. **b,** The weighted similarities of domains as predicted by deDoc (E) in the 23 chromosomes of GM06690 cells. **c,** Heatmap of the Hi-C data. The deDoc(E) predicted domains and compartment were highlighted in black and yellow sawteeth, respectively. The compartment A and B were not distinguished in this plot. **d,** The relative distance to the nearest compartment boundary from deDoc(E) predicted domain boundaries.

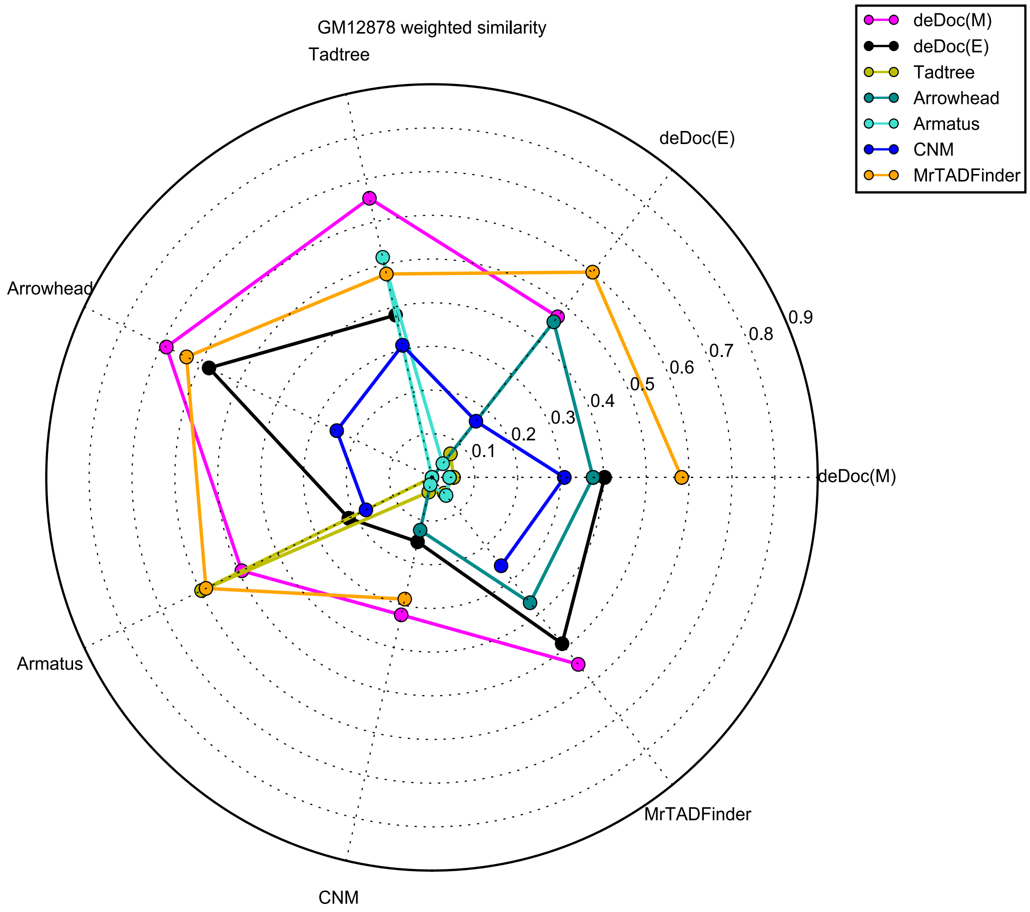

Supplementary Figure 2. The spider chart shows similarities between the TADs as predicted by different algorithms. Each spoke represents a group of comparison from a reference algorithm, indicated as the colored square, to the other algorithms. The values in the spokes are weighted similarities (WS). As each algorithm did not compare to itself, the curves are not closed. The data was about GM12878 from the Rao et al[4] .

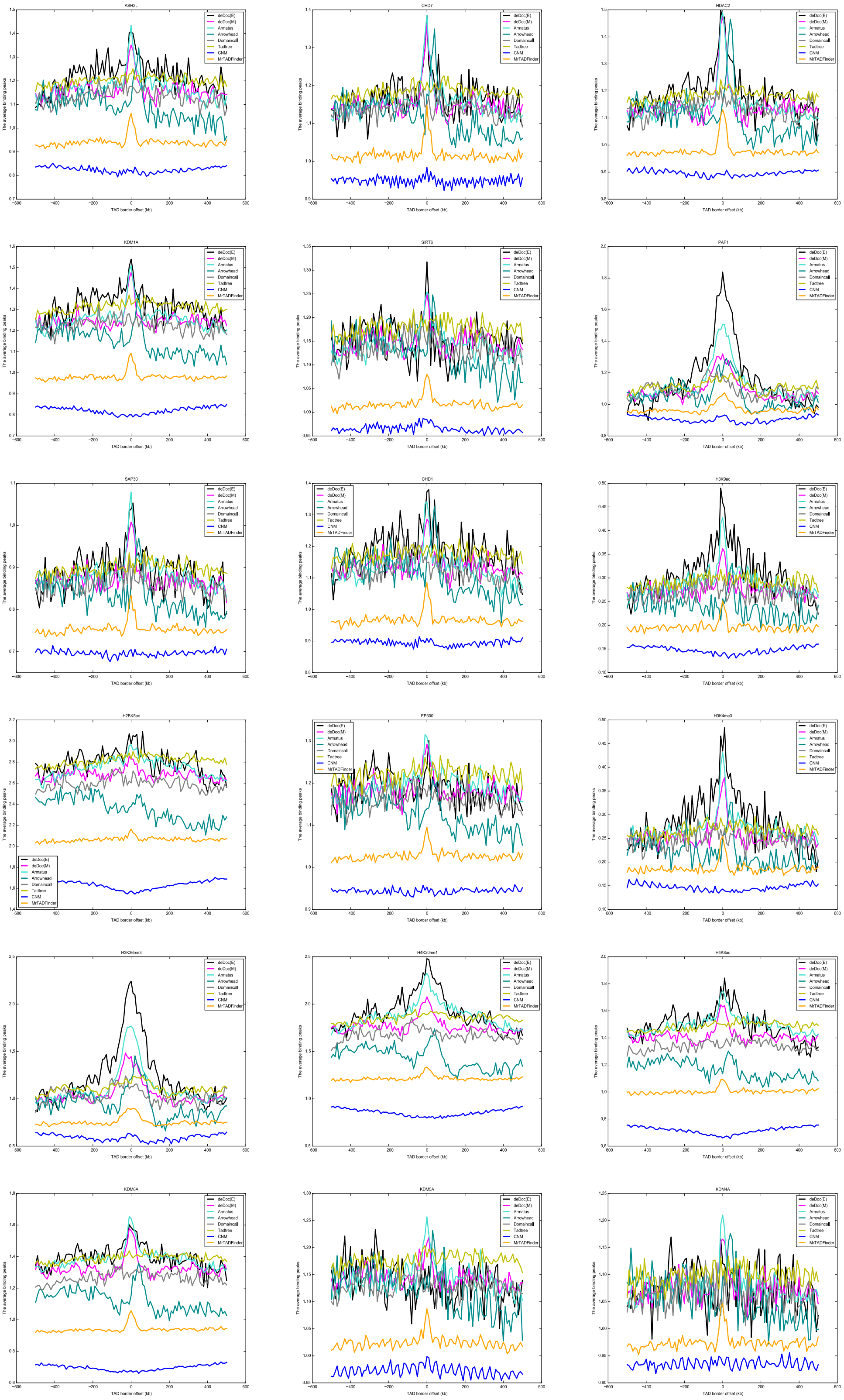
ASH2L
CHD7
HDAC2
KDM1A
SIRT6
PAF1
SAP30
CHD1
H3K9ac
H2BK5ac
EP300
H3K4me3
H3K36me3
H4K20me1
H4K8ac
KDM6A
KDM5A
KDM4A
The average binding peaks
TAD border offset (kb)
deDoc(E)
deDoc(M)
Armatus
Arrowhead
Domaincall
Tadtree
CNM
MrTADFinder

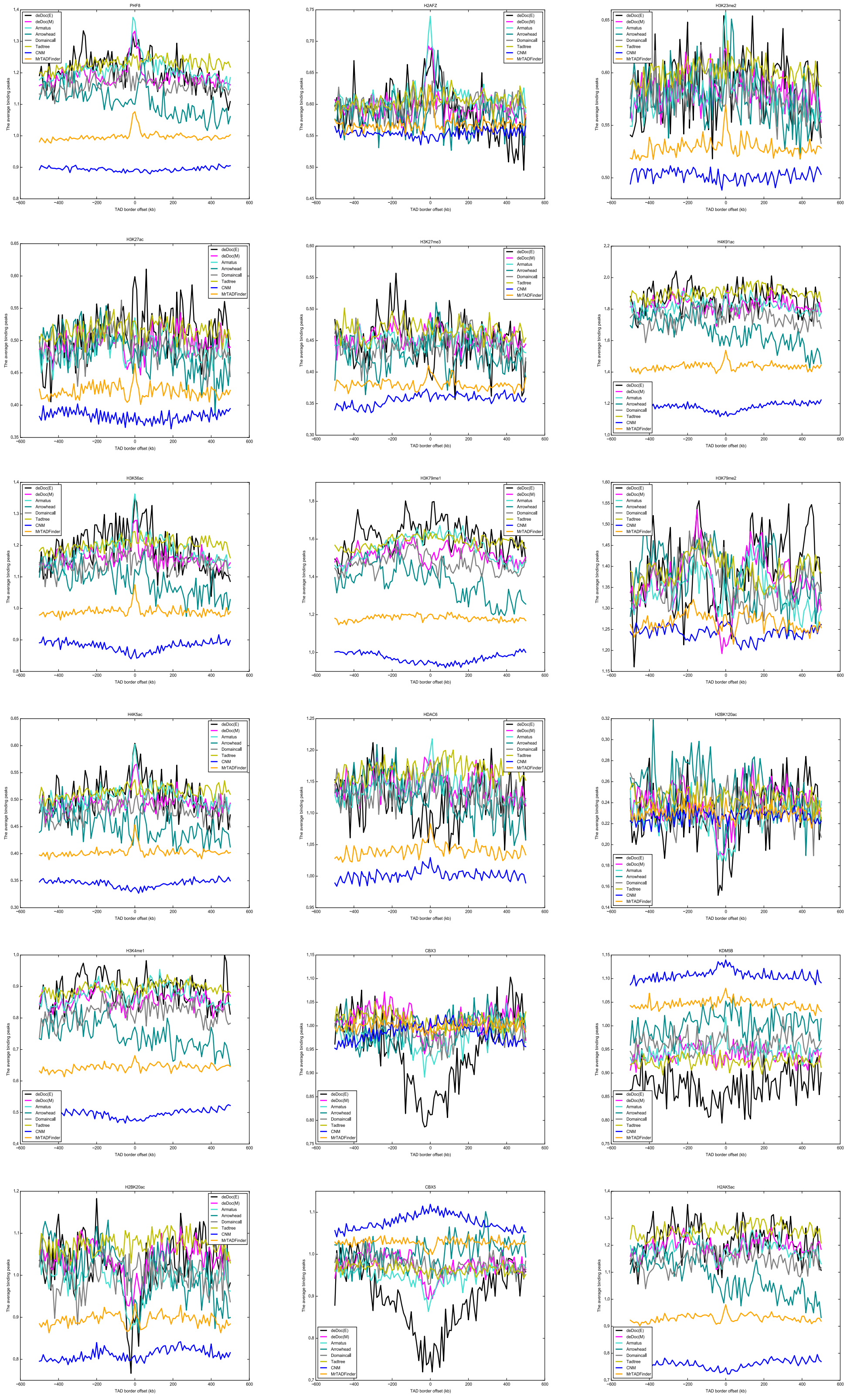
PHF8
H2AFZ
H3K23me2
H3K27ac
H3K27me3
H4K91ac
H3K56ac
H3K79me1
H3K79me2
H4K5ac
HDAC6
H2BK120ac
H3K4me1
CBX3
KDM5B
H2BK20ac
CBX5
H2AK5ac
deDoc(E)
deDoc(M)
Armatus
Arrowhead
Domaincall
Tadtree
CNM
MrTADFinder
The average binding peaks
TAD border offset (kb)

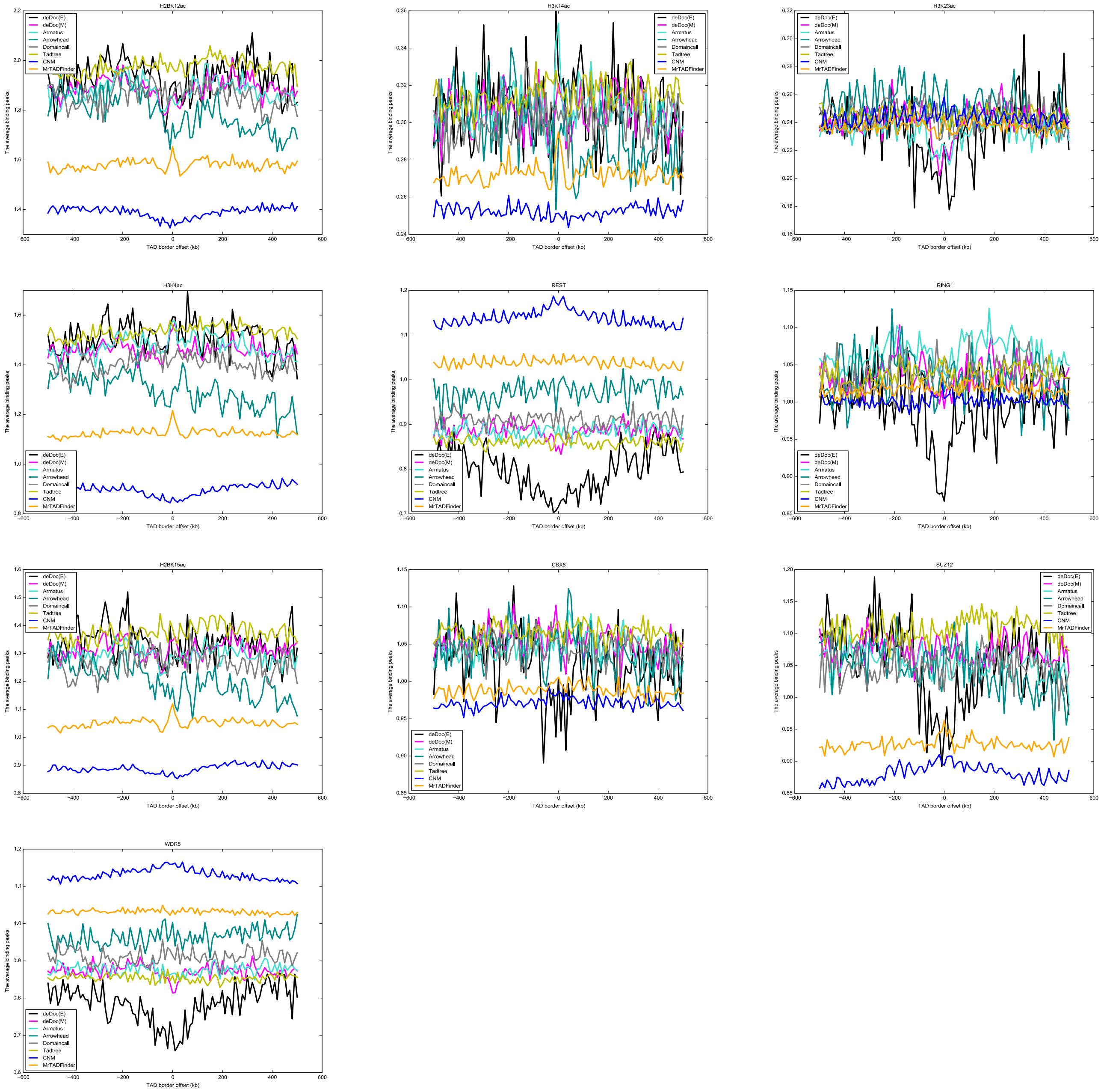

Supplementary Figure 3. deDoc identified border regions are enriched with some TF bindings. The Plot representing ChIP-seq peaks of TF enrichment from human ES cells (hES). Each curve represents a result from an algorithm. The data was from ENCODE project.

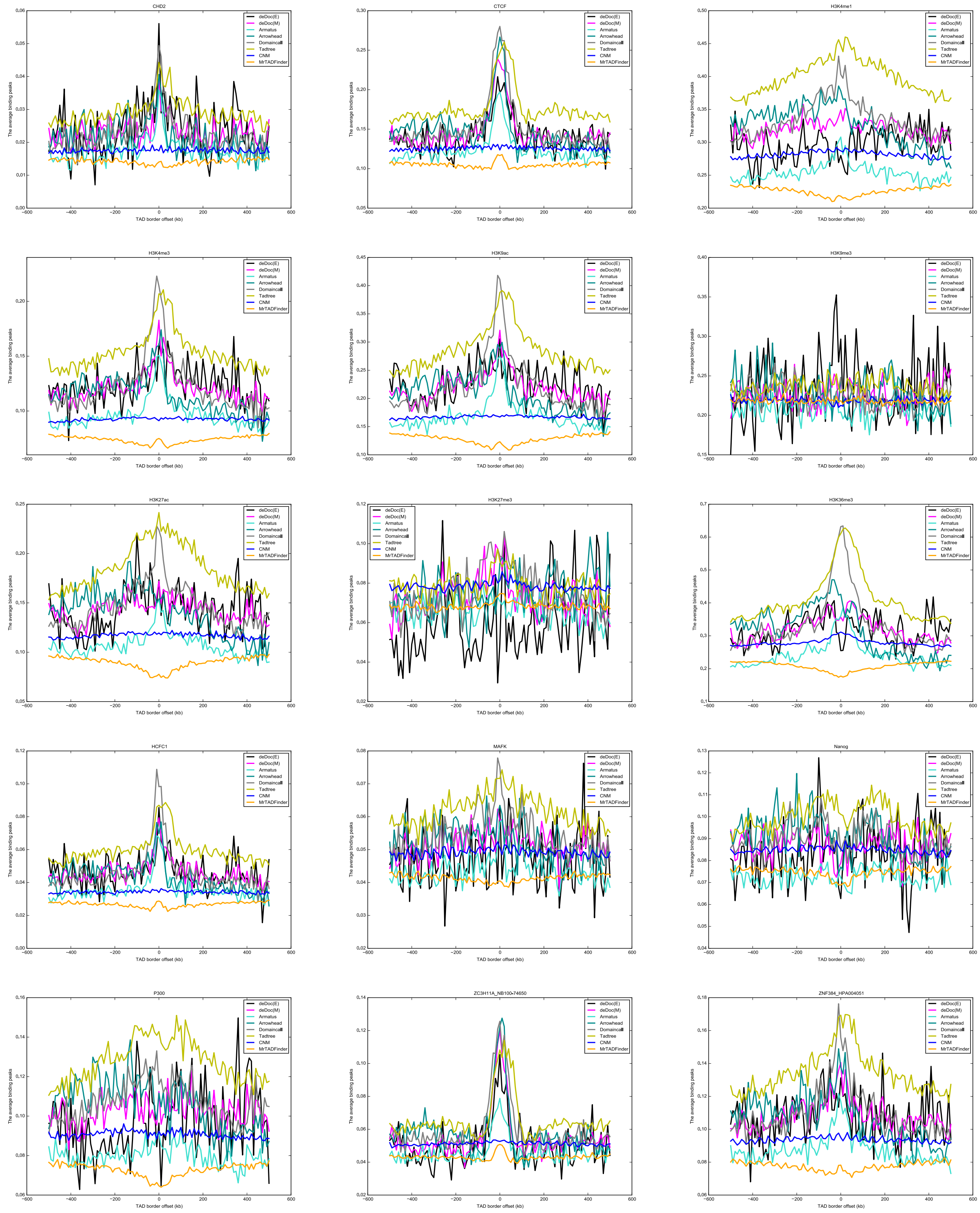

Supplementary Figure 4. deDoc identified border regions are enriched with some TF bindings. The Plot representing ChIP-seq peaks of TF enrichment from mouse ES cells (mES). Each curve represents a result from an algorithm. The data was from ENCODE project.

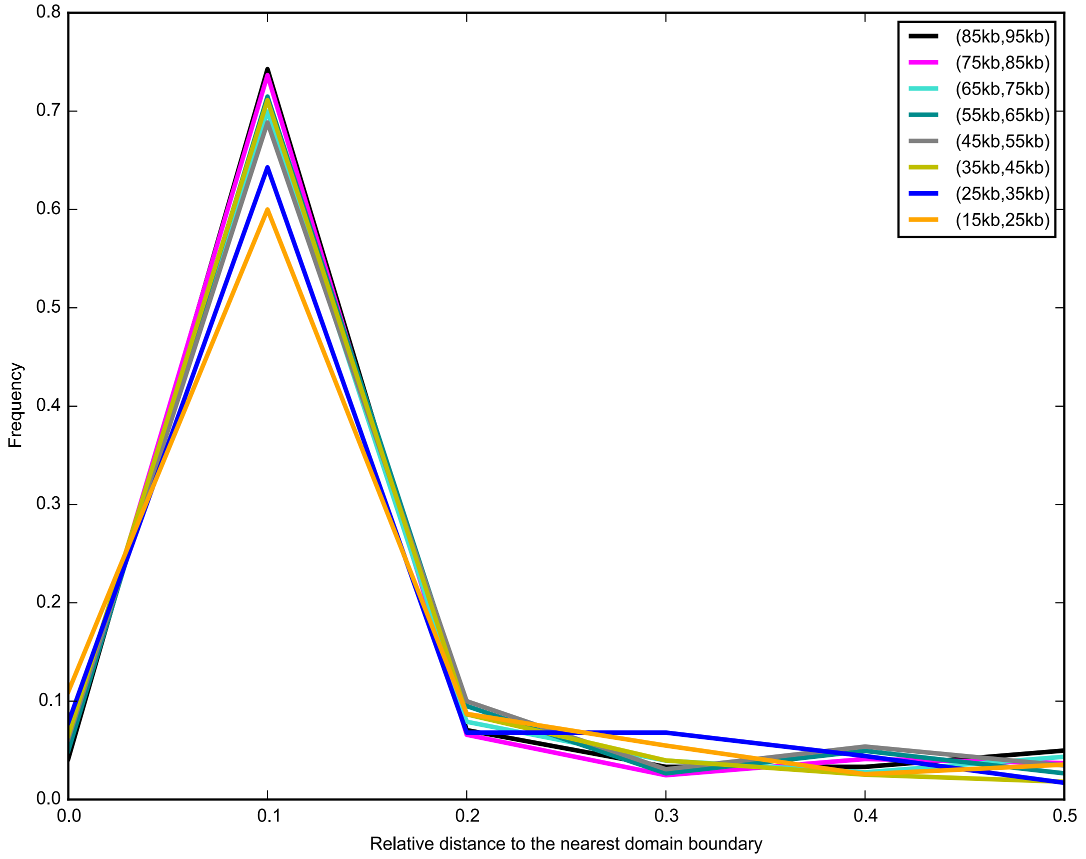

Supplementary Figure 5. The distribution of the relative distance to the nearest TAD borders using deDoc(M) with Dixon et al's data[7].

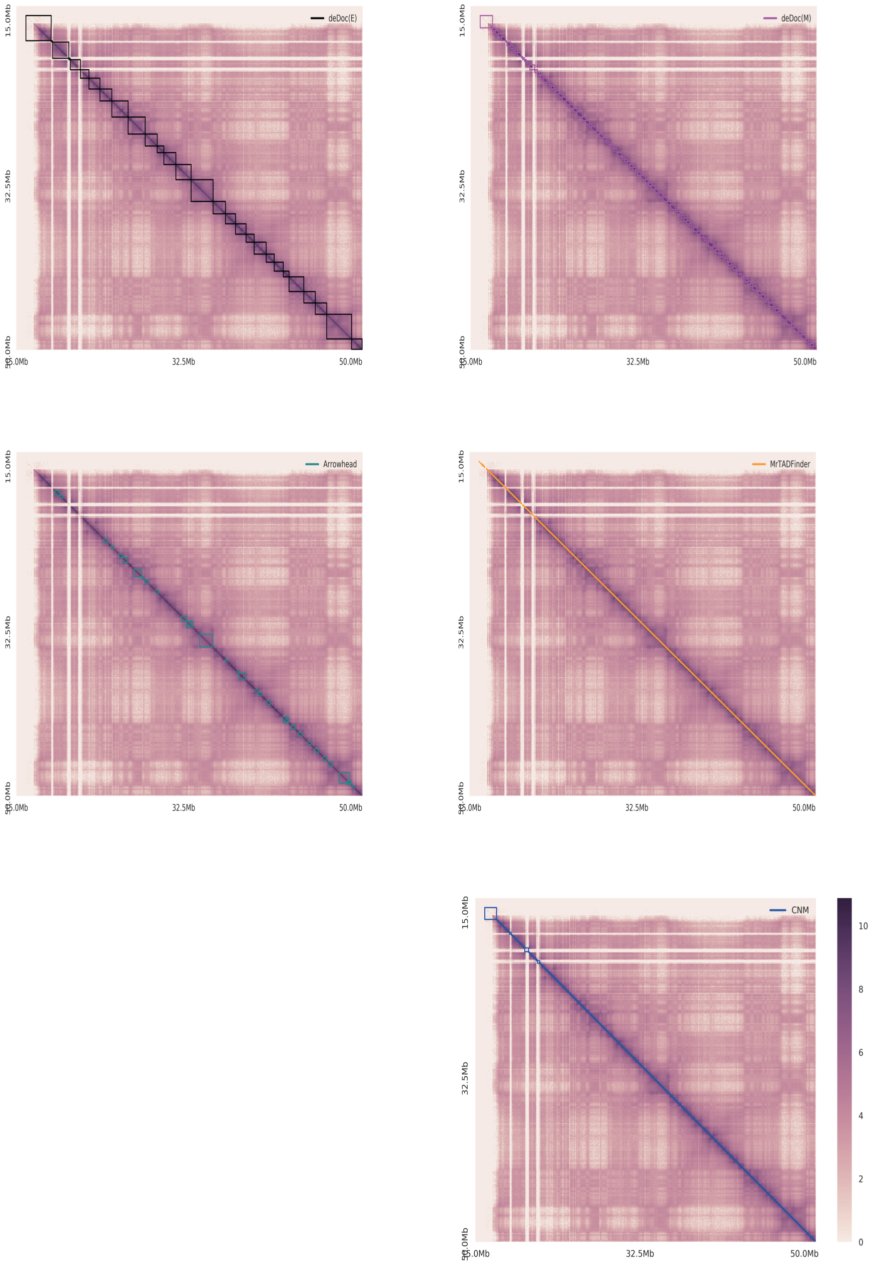

Supplementary Figure 6. The side-by-side comparison of the TADs as predicted by each algorithm using Rao et al's data[4] in chromosome 22.

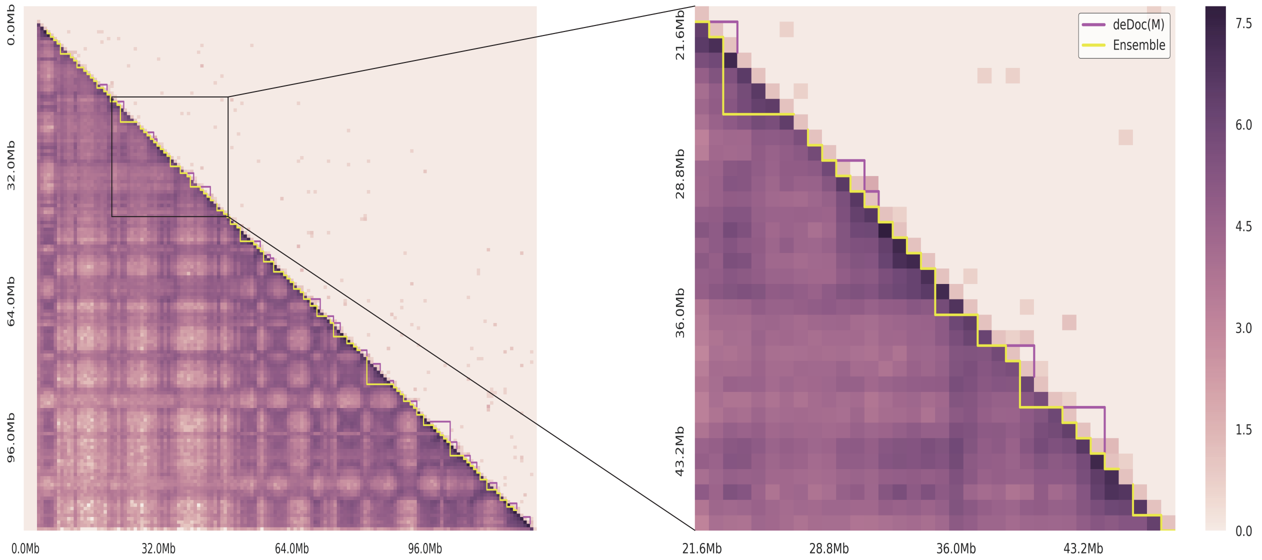

Supplementary Figure 7. Heatmap of ensemble and pooled Hi-C from a single cell. The deDoc(M) predicted domains and ensemble TADs were highlighted in magenta and yellow sawteeth, respectively.

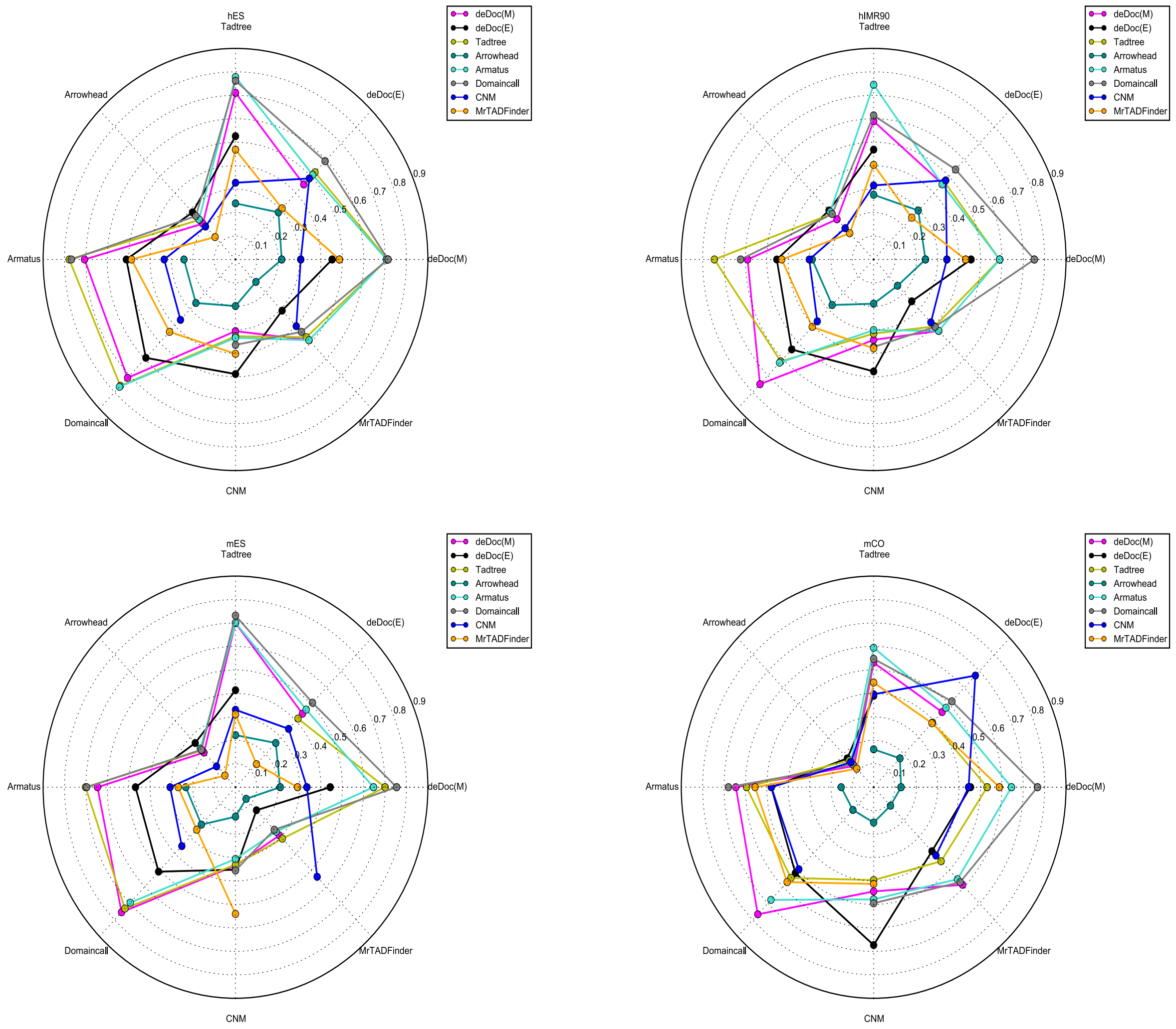

Supplementary Figure 8. Similarities as measured by *ws(P,Q)*, which is defined to be minimums of ws_Q^P and ws_P^Q.

Supplementary References

1. Li, A. & Pan, Y. Structural Information and Dynamical Complexity of Networks. *IEEE Transactions on Information Theory* **62**, 3290 - 3339 (2016).
2. Shannon, C.E. The lattice theory of information. *IEEE Transactions on Information Theory* **1**, 105-107 (1953).
3. Yaffe, E. & Tanay, A. Probabilistic modeling of Hi-C contact maps eliminates systematic biases to characterize global chromosomal architecture. *Nat Genet* **43**, 1059-65 (2011).
4. Rao, S.S. *et al.* A 3D Map of the Human Genome at Kilobase Resolution Reveals Principles of Chromatin Looping. *Cell* **159**, 1665-80 (2014).
5. Hu, M. *et al.* HiCNorm: removing biases in Hi-C data via Poisson regression. *Bioinformatics* **28**, 3131-3 (2012).
6. Forcato, M. *et al.* Comparison of computational methods for Hi-C data analysis. *Nat Methods* **14**, 679-685 (2017).
7. Dixon, J.R. *et al.* Topological domains in mammalian genomes identified by analysis of chromatin interactions. *Nature* **485**, 376-80 (2012).
8. Nagano, T. *et al.* Single-cell Hi-C reveals cell-to-cell variability in chromosome structure. *Nature* **502**, 59-64 (2013).
9. Lieberman-Aiden, E. *et al.* Comprehensive mapping of long-range interactions reveals folding principles of the human genome. *Science* **326**, 289-93 (2009).
10. Clauset, A., Newman, M.E. & Moore, C. Finding community structure in very large networks. *Phys Rev E Stat Nonlin Soft Matter Phys* **70**, 066111 (2004).
11. Filippova, D., Patro, R., Duggal, G. & Kingsford, C. Identification of alternative topological domains in chromatin. *Algorithms Mol Biol* **9**, 14 (2014).
12. Weinreb, C. & Raphael, B.J. Identification of hierarchical chromatin domains. *Bioinformatics* **32**, 1601-9 (2016).
13. Yan, K.K., Lou, S. & Gerstein, M. MrTADFinder: A network modularity based approach to identify topologically associating domains in multiple

resolutions. *PLoS Comput Biol* **13**, e1005647 (2017).
