# Supplementary material for: Decoding topologically associating domains with ultra-low resolution Hi-C data by graph structural entropy
Source: Nat Commun. 2018 Aug 15;9:3265. doi: 10.1038/s41467-018-05691-7 (PMC6093941; doi:10.1038/s41467-018-05691-7)
Supplement: Supplementary file 2 — Description of Additional Supplementary Files [file 41467_2018_5691_MOESM2_ESM.pdf]

Description of Additional Supplementary Files

File Name: Supplementary Data 1
Description: Predicted TADs.
